# New Marine-Inspired Oxadiazole Derivatives for Use Against Pancreatic Ductal Adenocarcinoma

**DOI:** 10.3390/md23080327

**Published:** 2025-08-14

**Authors:** Camilla Pecoraro, Daniela Carbone, Fares Hezam Mohammed Al Ostoot, Mahrou Vahabi, Giulia Lencioni, Patrizia Diana, Elisa Giovannetti, Barbara Parrino

**Affiliations:** 1 Dipartimento di Scienze e Tecnologie Biologiche Chimiche e Farmaceutiche (STEBICEF), Università Degli Studi di Palermo, Via Archirafi 32, 90123 Palermo, Italy; camilla.pecoraro@unipa.it (C.P.); fareshezammohammed.alostoot@unipa.it (F.H.M.A.O.); patrizia.diana@unipa.it (P.D.); barbara.parrino@unipa.it (B.P.); 2 Department of Medical Oncology, Cancer Center Amsterdam, Amsterdam UMC, VU University Medical Center (VUmc), De Boelelaan 1117, 1081 HV Amsterdam, The Netherlands; m.vahabi@amsterdamumc.nl (M.V.); e.giovannetti@amsterdamumc.nl (E.G.); 3 Cancer Pharmacology Laboratory, Fondazione Pisana per la Scienza, Via Ferruccio Giovannini 13, San Giuliano Terme, 56017 Pisa, Italy; g.lencioni@fpscience.it

**Keywords:** 1,2,4-oxadiazole, marine alkaloids, heterocycles, topsentin, pancreatic cancer, PDAC, antiproliferative, apoptosis, colony formation assay, drug resistance

## Abstract

Pancreatic ductal adenocarcinoma (PDAC) remains one of the deadliest malignancies, with limited effective therapeutic options due to late diagnosis, aggressive progression, and rapid development of drug resistance. In pursuit of novel treatments, this study reports the design, synthesis, and biological evaluation of a new series of topsentin derivatives, featuring a 1,2,4-oxadiazole core. The newly synthesized derivatives were screened for antiproliferative activity against multiple PDAC cell lines (SUIT-2, Patu-T, and PANC-1), identifying several compounds with potent growth-inhibitory effects, particularly on SUIT-2 and Patu-T cells. Further studies demonstrated that these compounds also significantly inhibited cell migration and reduced clonogenic potential, with IC_50_ values in the micromolar range. The results suggest that these marine-inspired 1,2,4-oxadiazole derivatives effectively target key hallmarks of PDAC, including proliferation, migration, and colony formation, supporting their further development as promising candidates for overcoming drug resistance and metastatic progression in pancreatic cancer.

## 1. Introduction

Pancreatic ductal adenocarcinoma (PDAC) is the predominant form of pancreatic cancer and is recognized as one of the most aggressive and lethal malignancies worldwide [1,2,3]. Despite advances in diagnostic imaging and surgical techniques, the prognosis for PDAC remains dismal, with a five-year survival rate lingering in the single digits. Surgical resection remains the only potentially curative intervention; however, the majority of patients present with advanced disease at diagnosis, rendering them ineligible for surgery. For these patients, systemic chemotherapy regimens such as FOLFIRINOX (a combination of folinic acid, fluorouracil, irinotecan, and oxaliplatin) or gemcitabine plus nab-paclitaxel constitute the current standard of care [4,5,6,7]. Unfortunately, these treatments are frequently associated with considerable toxicity, and their clinical efficacy is often compromised by the rapid emergence of drug resistance. The complex and heterogeneous nature of PDAC, including its dense stromal environment and intrinsic cellular plasticity, further complicates therapeutic intervention and underscores the urgent need for more effective and better-tolerated agents [8,9,10,11].

In the search for novel anticancer compounds, heterocyclic scaffolds have emerged as privileged structures in medicinal chemistry due to their unique physicochemical properties and broad spectrum of biological activities [12,13,14,15,16,17,18,19].

Marine natural products, particularly those derived from sponges, have proven to be a rich source of structurally unique and biologically potent compounds [20].

**Figure 1:**
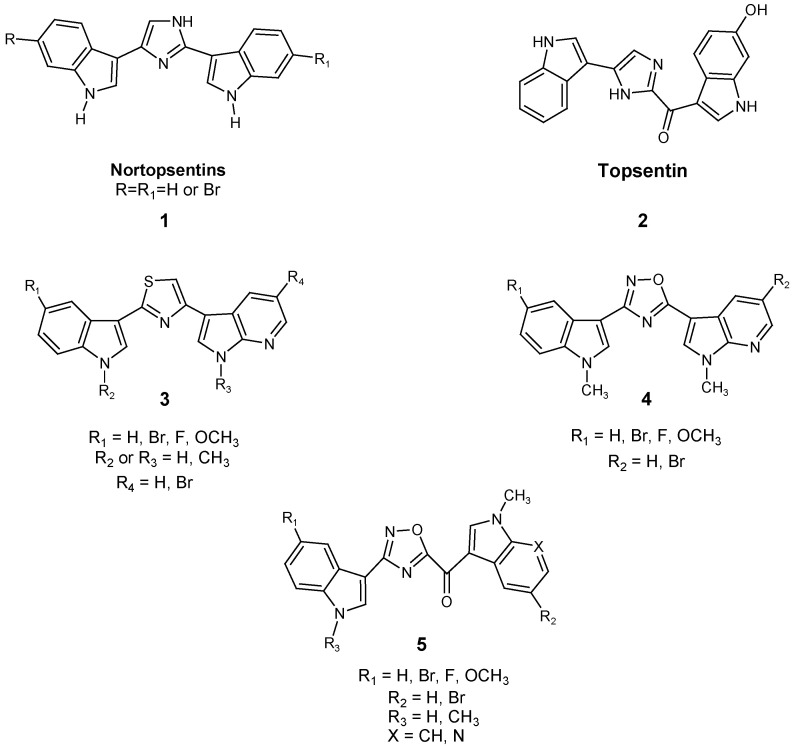
Chemical structures of nortopsentin **1** and topsentin **2** alkaloids and their derivatives **3**–**5**.

Nortopsentins **1** and topsentin **2** (Figure 1), bis-indolyl alkaloids isolated from deep-sea sponges (*Spongosorites ruetzler* and *Topsentia genitrix*), exemplify marine-derived chemotypes with potent antiproliferative activity [21,22]. These alkaloids share structural features common to many marine natural products, such as nitrogen-rich heterocycles, conjugated π-systems, and hydrogen-bonding motifs that facilitate interactions with biological targets. Produced by marine microorganisms as a means of chemical defense in extreme environments, these alkaloids display potent bioactivity, with IC_50_ values in the micromolar range against a broad spectrum of cancer models, including murine leukemia and human tumor cell lines [21,22].

Our group has systematically explored marine alkaloid-inspired architectures, focusing on replacing the central imidazole ring of nortopsentins with metabolically stable heterocycles while introducing azaindole substituents to enhance solubility. This approach yielded thiazole **3** and oxadiazole **4** derivatives (Figure 1) with improved cytotoxicity profiles (IC_50_: 0.64–0.89 μM) and synergistic interactions with standard chemotherapeutics [23,24].

Building on these findings, we turned to topsentin analogs, hypothesizing that their flexible carbonyl spacer could enable adaptive binding in PDAC’s complex stromal microenvironment. Previous work from our group has demonstrated the promising anticancer potential of topsentin analogs **5** featuring a 1,2,4-oxadiazole core replacing the natural imidazole ring (Figure 1). A series of these compounds was efficiently synthesized and screened against PDAC cell lines, exhibiting micromolar to submicromolar EC_50_ values and significant inhibition of cell migration. These effects, correlated with the modulation of epithelial-to-mesenchymal transition (EMT) markers such as SNAIL-1/2 and matrix metalloproteinase-9, were accompanied by enhanced apoptosis, as confirmed by flow cytometry. Additional analyses revealed activation of apoptotic pathways through the cleavage of caspase-3 and PARP and suggested involvement in intracellular signaling cascades relevant to PDAC progression [25].

**Figure 2:**
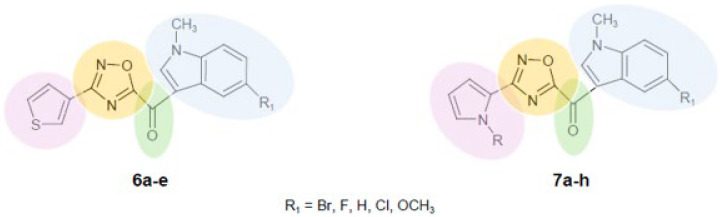
New oxadiazole-based topsentin derivatives **6–7**.

Encouraged by this finding and mindful of the importance of continuous discovery of novel derivatives to feed into the anticancer development pipeline, we continued exploring structure–activity relationships based on the “seed SAR” set [20,21] and aimed to achieve the following: (i) maintain the flexible carbonyl spacer of topsentin that may facilitate favorable interactions in biological systems; (ii) perform a bioisosteric replacement of an indole mojety with thiophene or pyrrole ring, which may modulate electron density and solubility [26]; (iii) evaluate the cytotoxic profile of series **6** and **7** against a panel of PDAC cell lines; and (iv) validate the mechanism of action of new 1,2,4-oxadiazole derivatives for selective targeting of cancer cells (Figure 2).

Therefore, in the present study, we describe the synthesis of a new series of topsentin-derived compounds featuring a 1,2,4-oxadiazole central core. Biological evaluation focuses on 2D and 3D PDAC cell line cytotoxicity and apoptosis induction, addressing both intrinsic resistance and stromal barriers. This work expands the repertoire of oxadiazole-based therapeutics for PDAC treatment.

## 2. Results and Discussion

### 2.1. Chemistry

**Scheme 1:**
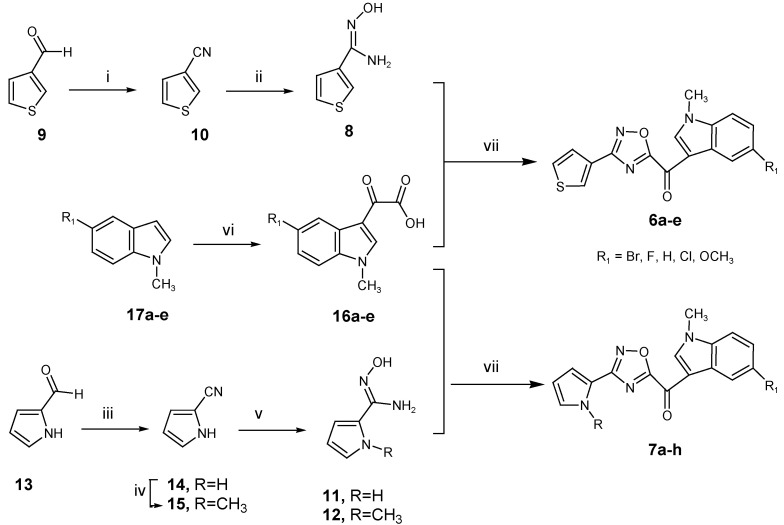
Synthesis of oxadiazole compounds **6a**–**e** and **7a**–**h**. Reagents and conditions: (i) iodine, ammonia solution (28% *v*/*v*), THF rt, 90 min, 98%; (ii) NH_2_OH·HCl, NaHCO_3_, ethanol, 30 min, then compound **10**, reflux, 5 h, 99%; (iii) NH_2_OH·HCl*, N*-methyl pyrrolidone, 115 °C, overnight, 99%; (iv) NaH 60% dispersion in mineral oil, 30 min, then CH_3_I, DMF, 4 h, 93%; (v) NH_2_OH·HCl, NaHCO_3_, ethanol, reflux, 5 h, 95–96%; (vi) oxalyl chloride, diethyl ether, 0 °C, 3 h, then 24 °C, 1 h, then NaOH 2 M, THF, rt, 12 h, 78–95%; (vii) EDC, HOBt, DMF, 0 °C, 15 min, then 100 °C, 15 min, 72–84%.

The synthesis of 1,2,4-oxadiazole derivatives **6** and **7** was achieved following the synthetic procedure reported in Scheme 1.

The key carboxamidine intermediate **8** was prepared from the commercial thiophene-3-carboxaldehyde **9**, which was treated with iodine (1.1 molar proportions) in ammonia water (28% solution) at room temperature, affording the desired nitrile **10** in very high yields (98%) [27]. The latter was dissolved in dry ethanol and reacted with sodium hydrogen carbonate and hydroxylamine hydrochloride (NH_2_OH·HCl), allowing the synthesis of derivative **8** in good yields (99%) [28].

The carboxamidine intermediates **11** and **12** were prepared from commercial pyrrole-3-carboxaldehyde **13**, which was dissolved in dry *N*-methyl pyrrolidone and reacted with NH_2_OH·HCl. The reaction, heated at 115 °C, allowed the synthesis of derivative **14** in a good yield (99%). The latter was converted into its methylated derivative **15**, using sodium hydride as a base and iodomethane (CH_3_I) as a methylating agent. The cyano derivative **14** or the methyl derivative **15** were then dissolved in dry ethanol and reacted with sodium hydrogen carbonate and hydroxylamine hydrochloride (NH_2_OH·HCl), allowing the synthesis of derivatives **11** or **12**, respectively, in good yields (95–96%).

The key intermediates, (1-methyl-1*H*-pyrrolo [2,3-b]pyridine-3-yl)-oxo-acetic acid **16a**–**e**, were synthesized as previously described [25] by treating methyl indole precursor **17** with oxalyl chloride at 0 °C, and we then converted the acyl chlorides into oxo-acetic acids (78–95%) using a sodium hydroxide (NaOH) solution at room temperature.

The reaction between the key building blocks **8** or **11,12** with **16a**–**e** was performed in DMF and in the presence of *N*-(3-dimethylaminopropyl)-*N*′-ethylcarbodiimide hydrochloride (EDC) and hydroxybenzotriazole (HOBt) as the coupling reagents, inducing the formation of an amide bond by previous activation of the carboxylic acid group. Subsequent in situ temperature-catalyzed cyclodehydration, warming the reaction mixture at 100 °C, gave the desired oxadiazole derivatives **6a**–**e** and **7a**–**h** in yields ranging from 72–84% (Table 1).

### 2.2. Biological Studies

#### 2.2.1. Antiproliferative Activity of the New 1,2,4-Oxadiazole Compounds **6a**–**e** and **7a**–**h** Against PDAC Cells

**Figure 3:**
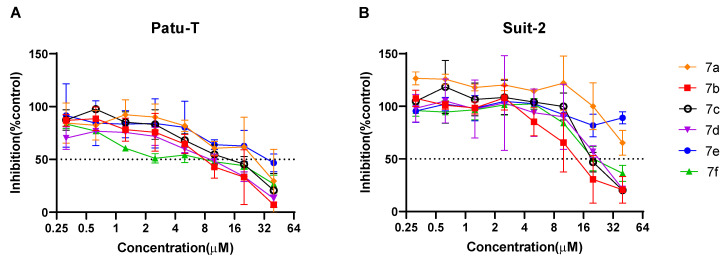
Effects of the most active compounds **7a**–**f** on the viability of Patu-T (**A**) and Suit-2 (**B**) PDAC cell lines. Cells were treated with nine different concentrations of the compounds and cell survival was measured after 72 h by SRB assay in comparison to untreated control cells. Values are reported as the mean ± SEM of three separate experiments, performed in triplicate.

All the oxadiazole topsentin compounds were screened across two immortalized PDAC cell lines (SUIT-2 and Patu-T) to identify compounds with the most pronounced growth-inhibitory effects (Figure 3).

Among the assayed compounds, derivatives **7f**, **7d,** and **7b** demonstrated the highest potency towards Patu-T cells, with GI_50_ values of 5.46 µM, 6.86 µM, and 7.07 µM, respectively. In contrast, the same compounds showed considerably weaker activity against SUIT-2 cells, with GI_50_ values ranging widely from 16.53 to 223.10 µM. This >30-fold difference in sensitivity between the two cell lines suggests underlying biological variations, such as differences in drug uptake, metabolism, or target expression, which may influence the compounds’ efficacy.

#### 2.2.2. Antimigratory Activity

Given that early metastasis behavior is a hallmark of PDAC and a major factor contributing to its poor therapeutic response and unfavorable prognosis [29,30], our study aimed to evaluate the antimigratory potential of newly developed oxadiazole topsentin derivatives.

Compounds **7b**–**f**, which demonstrated the most potent antiproliferative effects against the PDAC cell lines, were selected for further evaluation of their impact on cell migration using scratch wound healing assays in SUIT-2 and Patu-T cells.

**Figure 4:**
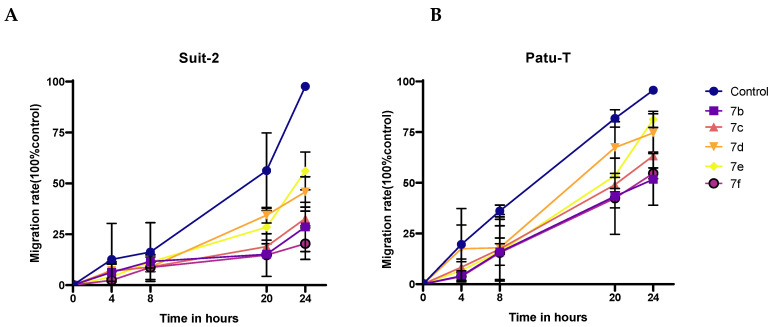

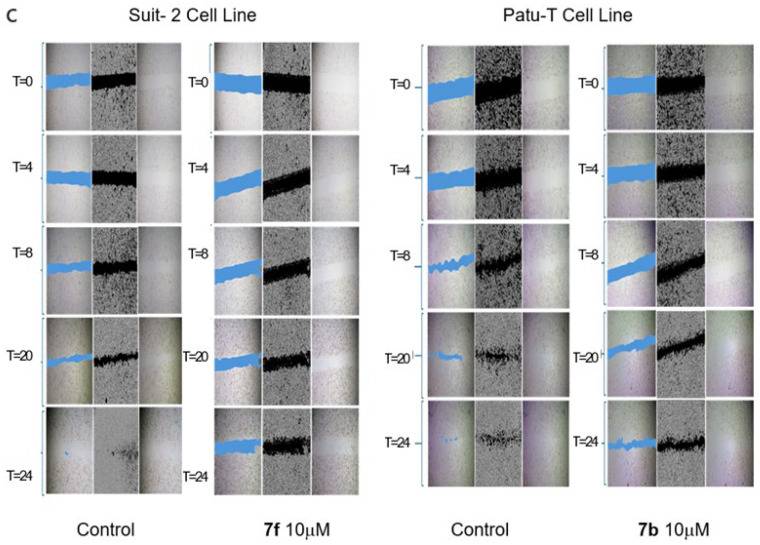
Modulation of the migration rate in PDAC cells. Charts express percentages of migration monitored over time (0, 4, 8, 20, and 24 h) of Suit-2 (**A**) or Patu-T (**B**) cells treated with the most active compounds **7b**–**f**. (**C**) Representative images from wound healing assay of Suit-2 and Patu-T cell cultures treated with or without compound **7f** and **7b**, respectively.

The antimigratory activity of compounds **7b**–**f** in the SUIT-2 cell line was assessed at 10 μM via scratch wound healing assays, with migration rates measured at 0, 8, 20, and 24 h and compared to the controls. The control group exhibited the highest migration rate, indicative of robust cell motility. All the tested compounds significantly inhibited cell migration, underscoring their potential utility in targeting metastatic PDAC (Figure 4). Notably, derivative **7f** showed the most significant antimigratory activity, with a migration rate of 20%, reflecting an 80% reduction in migration compared to the controls. Similarly, compounds **7b**, **7c**, and **7d** exhibited a reduction in migration in the range of 67–72% compared to the controls. Meanwhile, compound **7e** demonstrated the weakest antimigratory effect, with a migration rate of 44% relative to the controls (Figure 4A).

In the case of the Patu-T cell line, among the tested compounds, derivatives **7b** and **7f** displayed the strongest antimigratory activity, with, respectively, a 48.30% and 45.34% reduction in migration compared to the controls (Figure 4B). A good antimigratory activity was also observed for compounds **7c** and **7d,** which showed a migration rate of 36.71% and 25.30%, respectively (Figure 4B).

These findings collectively highlight the capacity of oxadiazole topsentin derivatives, particularly **7f** and 7**b** (Figure 4C), to inhibit PDAC cell migration, supporting their further development as therapeutic agents targeting metastatic progression.

#### 2.2.3. Clonogenic Assay

To confirm the anticancer activity of potent compounds, the colony formation assay was performed. Since early metastasis and therapeutic resistance are hallmarks of PDAC, reducing the clonogenic potential of cancer cells is a key strategy in preventing tumor progression [31,32].

**Figure 5:**
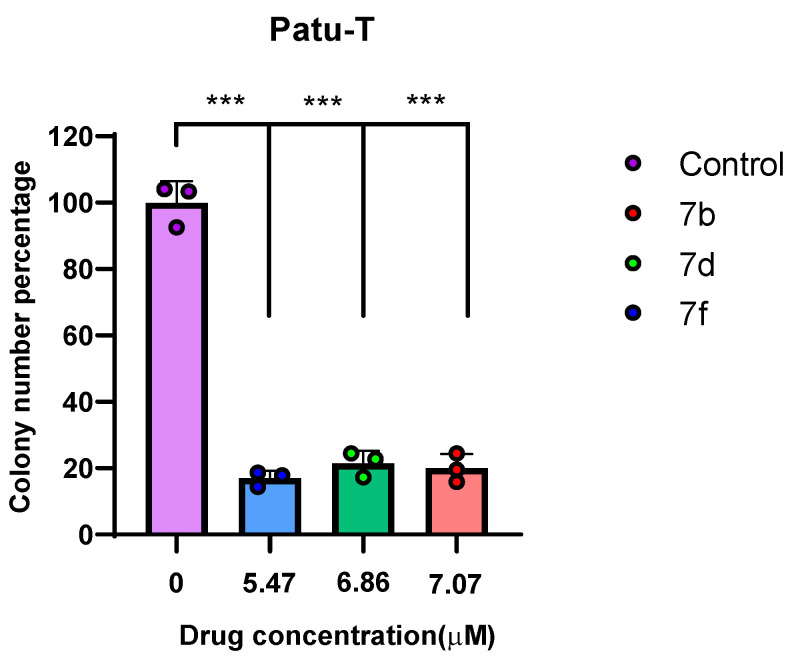
Colony formation assay in Patu-T cell line treated with compounds **7b**, **7d**, and **7f** at their respective IC_50_ concentrations. *p* values were calculated with student’s *t*-test. *** *p* < 0.001.

The results, illustrated in the bar graph in Figure 5, show the effects of compounds **7b**, **7d**, and **7f** at their respective IC_50_ concentrations (7.07 µM for **7b**, 6.86 µM for **7d**, and 5.47 µM for **7f**) on the colony-forming ability of the Patu-T cells compared to the untreated controls.

The colony formation assay measures the ability of cancer cells to survive and proliferate over time, with a lower percentage of colony formation indicating greater efficacy in inhibiting long-term cell survival. Treatment with compound **7b** resulted in a significant reduction in colony formation to approximately 19.93% of that of the controls, corresponding to an 80.07% inhibition of clonogenic potential. Similarly, compound **7d** reduced colony formation to about 21.46%, representing a 78.54% decrease, demonstrating strong but slightly less potent inhibition than **7b**. Among the tested compounds, **7f** exhibited the most pronounced effect, decreasing colony formation to 16.96%, which translates to an 83.04% reduction in clonogenic capacity. These findings highlight compounds **7b**, **7d**, and **7f** as the most effective inhibitors of long-term survival and proliferation in Patu-T cells, reinforcing their potential as lead candidates for further anticancer development.

#### 2.2.4. Cell Apoptosis Analysis

The apoptosis assay was performed to evaluate the capacity of the potent compounds **7b**, **7d,** and **7f** to induce programmed cell death in Patu-T pancreatic cancer cells. Apoptosis, or programmed cell death, a tightly regulated process of cellular self-destruction, represents a critical mechanism through which anticancer agents eliminate malignant cells. In PDAC, resistance to apoptosis is a major factor contributing to aggressive tumor growth, metastasis, and poor response to therapy [33]. Therefore, identifying compounds capable of effectively triggering apoptosis in PDAC cells is essential for developing new therapeutic strategies.

**Figure 6:**
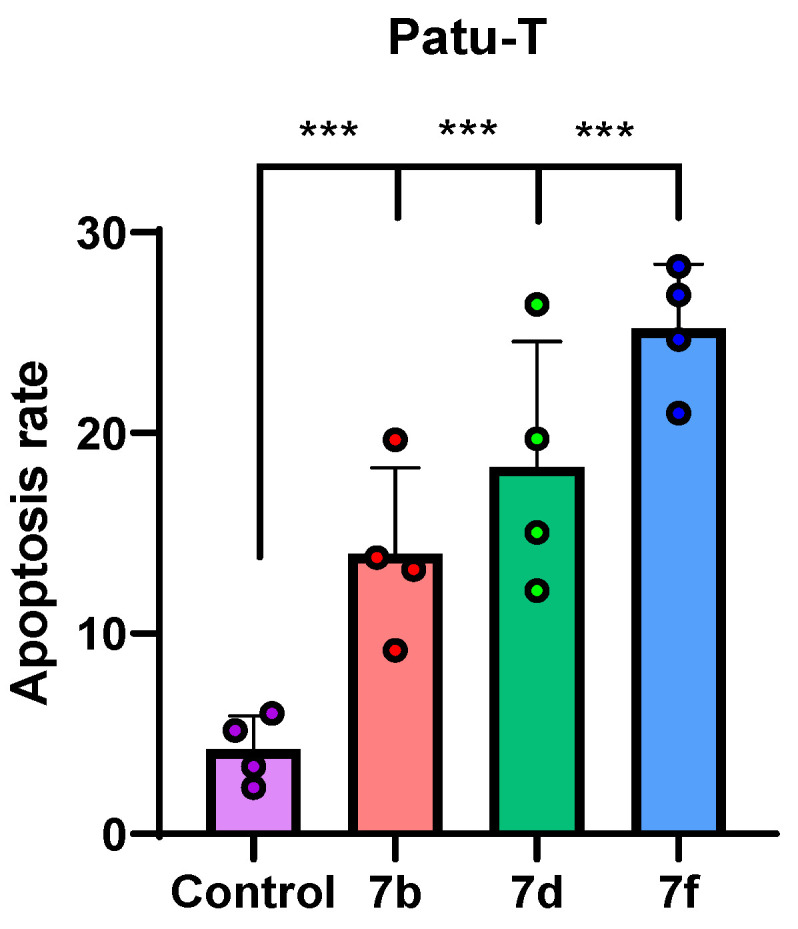
Apoptosis rate induced by compounds **7b**, **7d**, and **7f** in the Patu-T cell line compared to the untreated controls. *p* values were calculated with student’s *t*-test. *** *p* < 0.001.

The results, depicted in the accompanying bar graph in Figure 6, demonstrate that all three compounds significantly increased apoptosis rates compared to the untreated controls. Among them, compound **7f** exhibited the strongest pro-apoptotic effect, inducing apoptosis in 25.21% of the Patu-T cells, corresponding to a 5.2-fold increase relative to the controls. Compound **7d** also elicited a notable apoptotic response, with an apoptosis rate of 18.32%, representing a 4.8-fold increase. Compound **7b** induced apoptosis in 13.95% of cells, corresponding to a 4.4-fold increase over the controls. These findings confirm the ability of oxadiazole topsentin derivatives, particularly compound **7f**, to effectively promote programmed cell death in PDAC cells, supporting their potential as promising anticancer agents.

#### 2.2.5. ADME Prediction

**Figure 7:**
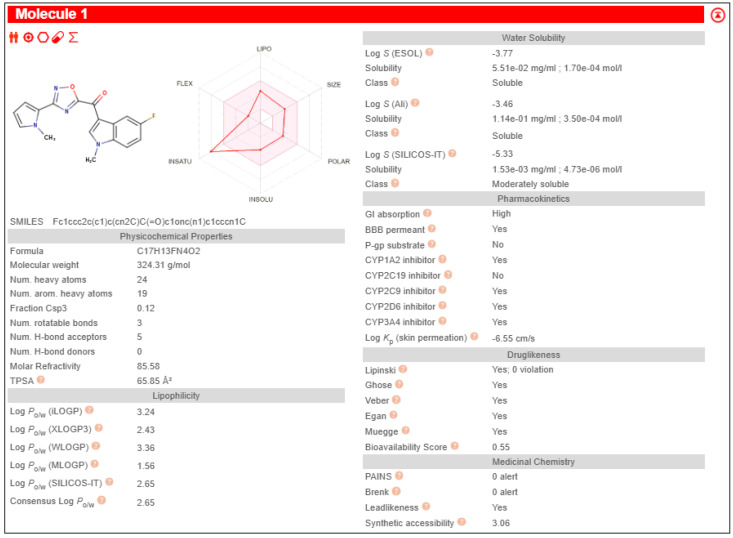
ADME prediction of compound **7f**.

In order to evaluate and predict the fate of compound 7**f** within the human body, ADME and pharmacokinetic predictions were carried out using freely available in silico SwissADME software (http://www.swissadme.ch/, accessed on 28 July 2025) [34]. Compound **7f**, as shown in Figure 7, displayed promising physicochemical and pharmacokinetic parameters in ADME prediction studies, showing 5 H-bond acceptors, 0 H-bond donors, 3 rotatable bonds, and a topological polar surface area (TPSA) of 65.58 Å^2^.

Lipophilicity is estimated by multiple log P methods, with a consensus log *P_o/w_* of 2.65, indicating moderate lipophilicity. Moreover, our compound exhibits moderate aqueous solubility, with Log S values (ESOL, Ali, SILICOS-IT) ranging between −3.77 and −3.46, classifying it as soluble to moderately soluble. Pharmacokinetic predictions suggest high gastrointestinal absorption, P-glycoprotein substrate properties, and the ability to inhibit several cytochrome P450 isoforms (CYP1A2, CYP2C9, CYP3A4), but not CYP2C19 or CYP2D6. The molecule is predicted to cross the blood–brain barrier and has a skin permeability log *K_p_* of −6.56 cm/s. Drug-likeness assessments show no violations of Lipinski’s, Veber’s, or other rules, with a bioavailability score of 0.55.

## 3. Materials and Methods

### 3.1. Chemistry

The anhydrous solvents utilized in organic synthesis (acetonitrile, dimethylformamide, and diethyl ether) and the reagents were purchased from Sigma-Aldrich Co., Alfa Aesar, VWR International, and Acros Organics. Additional solvents were purified and dried via conventional techniques, such as toluene, which was distilled from calcium hydride, while ethanol was refined using iodine and magnesium. All anhydrous solutions were maintained over 4 Å molecular sieves. All reactions sensitive to air or moisture were conducted using oven-dried glassware in an inert dry nitrogen environment. Analytical thin layer chromatography (TLC) was conducted on silica gel 60 F254 plates with a thickness of 0.25 mm, and the produced plates were analyzed under ultraviolet (UV) light. All melting points were determined using a Buchi–Tottoly capillary device and were uncorrected. ^1^H and ^13^C NMR spectra were acquired at 200 and 50 MHz, respectively, in DMSO-*d_6_* solution, using a Bruker Avance II series 200 MHz spectrometer. Chemical shifts are indicated in parts per million (δ), coupling constants (J) are quantified in Hertz (Hz), and splitting patterns are classified as singlet (s), doublet (d), triplet (t), quartet (q), multiplet (m), doublet of doublets (dd), and triplet of doublets (td). Column chromatography was conducted using MERK silica gel 230–400 mesh ASTM.

#### 3.1.1. General Procedure for the Synthesis of Thiophene-3-Carbonitrile **10**

A solution of the commercial thiophene-3-carboxaldehyde **9** (1.0 g, 8.90 mmol) in anhydrous THF (6.7 mL) was treated with iodine (2.37 g, 9.3 mmol, 1.1 molar proportions) in ammonia water (28% solution) (27 mL). The reaction mixture was stirred at room temperature for 1 h and 30 min and was then extracted with ethyl acetate (3 × 20 mL), dried (Na_2_SO_4_), and filtered and the solvent evaporated under reduced pressure. The residue was purified by column chromatography using cyclohexane as eluent.

Yield: 98%; brown oil; ^1^H NMR (200 MHz, DMSO-*d_6_*) δ: 7.51 (1H, dd, *J* = 5.1, 1.2 Hz), 7.82 (1H, dd, *J* = 5.1, 2.9 Hz), 8.57 (1H, dd, *J* = 2.9, 1.2 Hz); ^13^C{^1^H} NMR (50 MHz, DMSO-*d_6_*) δ: 115.4, 125.5, 126.1, 126.9, 131.9; Anal. Calculated for C_5_H_3_NS (MW: 109.15): C, 55.02; H, 2.77; N, 12.83%. Found: C, 55.09; H, 2.85; N, 12.88%.

#### 3.1.2. General Procedure for the Synthesis of N-Hydroxy-Thiophene-3-Carboxamidine **8**

To a solution of hydroxylamine hydrochloride (NH_2_OH·HCl) (0.77 g, 11.09 mmol) in dry ethanol was added sodium hydrogen carbonate (1.3 g, 15.11 mmol) and the mixture was stirred at room temperature for 30 min. After that, thiophene-3-carbonitrile **10** (1.1 g, 10.07 mmol) was added in portions and the reaction mixture was stirred at reflux for 5 h. After cooling, the solvent was evaporated under reduced pressure, the mixture was poured into water and ice, and the obtained precipitate was filtered off and dried, to give the desired product **8**.

Yield: 99%; white solid; ^1^H NMR (200 MHz, DMSO-*d_6_*) δ: 5.75 (2H, s), 7.32 (1H, dd, *J* = 5.0, 1.0 Hz), 7.49 (1H, dd, *J* = 5.0, 2.9 Hz), 7.79 (1H, dd, *J* = 2.9, 1.0 Hz), 9.45 (1H, s); ^13^C{^1^H} NMR (50 MHz, DMSO-*d_6_*) δ: 125.5, 126.1, 126.9, 131.9, 160.8; Anal. Calculated for C_5_H_6_N_2_OS (MW: 142.18): C, 42.24; H, 4.25; N, 19.70%. Found: C, 42.31; H, 4.32; N, 19.63%.

#### 3.1.3. General Procedure for the Synthesis of 1H-Pyrrole-2-Carbonitrile **14**

Commercial pyrrole-3-carboxaldehyde **13** (2 g, 21.03 mmol) was dissolved in dry N-methyl pyrrolidone, reacted with hydroxylamine hydrochloride (NH_2_OH·HCl) (1.75 g, 25.24 mmol), and heated at 115 °C overnight. After cooling, the resulting mixture was extracted with dichloromethane (2 × 10 mL), dried (Na_2_SO_4_), and filtered and the solvent was evaporated under reduced pressure. The residue was purified by column chromatography using cyclohexane/ethyl acetate 98:2 as eluent.

Yield: 99%; brown oil; ^1^H NMR (200 MHz, DMSO-*d_6_*) δ: 6.21 (1H, dd, *J* = 3.5, 2.3 Hz), 6.90 (1H, d, *J* = 3.5 Hz), 7.14 (1H, d, *J* = 2.3 Hz), 12.28 (s, 1H); ^13^C{^1^H} NMR (50 MHz, DMSO-*d_6_*) δ: 100.0, 110.0, 115.3, 120.0, 125.1; Anal. Calculated for C_5_H_4_N_2_ (MW: 92.10): C, 65.21; H, 4.38; N, 30.42%. Found: C, 65.33; H, 4.42; N, 30.48%.

#### 3.1.4. General Procedure for the Synthesis of 1-Methyl-1H-Pyrrole-2-Carbonitrile **15**

To a solution of 1*H*-pyrrole-2-carbonitrile **14** (2.18 g, 23.74 mmol) in anhydrous DMF (50 mL), sodium hydride 60% dispersion in mineral oil (1.14 g, 47,48 mmol) was added. After stirring at ambient temperature for 30 min, the mixture was treated with methyl iodide (1.75 mL, 47.48 mmol). Then, the resulting suspension was stirred at room temperature for 4 h. The reaction mixture was treated with water (15 mL) and extracted with ethyl acetate (3 × 15 mL), dried (Na_2_SO_4_), and evaporated under reduced pressure. The product obtained was purified by column chromatography, using cyclohexane/ethyl acetate 9:1 as eluent.

Yield: 93%; brown oil; ^1^H NMR (200 MHz, DMSO-*d_6_*) δ: 3.74 (3H, s), 6.16 (1H, dd, *J* = 4.0, 2.6 Hz), 6.90 (1H, dd, *J* = 4.0, 1.6 Hz), 7.11–7.22 (1H, m); ^13^C{^1^H} NMR (50 MHz, DMSO-*d_6_*) δ: 35.4, 103.5, 109.5, 114.3, 120.3, 129.4; Anal. Calculated for C_6_H_6_N_2_ (MW: 106.13): C, 67.90; H, 5.70; N, 26.40%. Found: C, 67.82; H, 5.63; N, 26.36%.

#### 3.1.5. General Procedure for the Synthesis of N-Hydroxy-1H-Pyrrole-2-Carboxamidine (**11**) or N-Hydroxy-1-Methyl-1H-Pyrrole-2-Carboxamidine (**12**)

To a solution of hydroxylamine hydrochloride (NH_2_OH·HCl) (0.77 g, 11.09 mmol) in dry ethanol, sodium hydrogen carbonate was added (1.3 g, 15.11 mmol) and stirred at room temperature for 30 min. After that, 1*H*-pyrrole-2-carbonitrile **14** or 1-methyl-1*H*-pyrrole-2-carbonitrile **15** (1.1 g, 10.07 mmol) were added in portions and the reaction mixture was stirred at reflux for 5 h. After cooling, the solvent was evaporated under reduced pressure and the mixture was extracted with ethyl acetate (3 × 15 mL), dried (Na_2_SO_4_), and evaporated under reduced pressure. The product obtained was further purified by column chromatography, using cyclohexane/ethyl acetate 1:1 as eluent.

##### N-Hydroxy-1H-Pyrrole-2-Carboxamidine (**11**)

Yield: 96%; white solid; ^1^H NMR (200 MHz, DMSO-*d_6_*) δ: 5.75 (2H, s), 6.00 (1H, dd, *J* = 3.9, 2.5 Hz), 6.43 (1H, d, *J* = 3.9 Hz), 6.68 (1H, d, *J* = 2.5 Hz), 9.13 (1H, s), 10.82 (s, 1H); ^13^C{^1^H} NMR (50 MHz, DMSO-*d_6_*) δ: 107.4, 108.10, 119.5, 125.0, 146.8; Anal. Calculated for C_5_H_7_N_3_O (MW: 125.13): C, 47.99; H, 5.64; N, 33.58%. Found: C, 48.02; H, 5.67; N, 33.61%.

##### N-Hydroxy-1-Methyl-1H-Pyrrole-2-Carboxamidine (**12**)

Yield: 95%; white solid; ^1^H NMR (200 MHz, DMSO-*d_6_*) δ: 3.70 (3H, s), 5.53 (2H, s), 5.96 (1H, dd, *J* = 3.7, 2.7 Hz), 6.38 (1H, dd, *J* = 3.7, 1.9 Hz), 6.61–6.85 (1H, m), 9.38 (1H, s); ^13^C{^1^H} NMR (50 MHz, DMSO-*d_6_*) δ: 36.9, 107.0, 110.0, 125.6, 125.9, 147.3; Anal. Calculated for C_6_H_9_N_3_O (MW: 139.16): C, 51.79; H, 6.52; N, 30.20%. Found C, 51.78; H, 6.51; N, 30.19%.

#### 3.1.6. General Procedure for the Synthesis of (1-methyl-1H-indol-3-yl)-oxo-Acetic Acids (**16a**–**e**)

To a solution of the opportune methyl indole of the type **17** (10 mmol) in anhydrous diethyl ether (20 mL), oxalyl chloride (11.16 mmol, 0.95 mL) was added dropwise at 0 ° C. The reaction mixture was left to stir at 0 ° C for 3 h and then brought to room temperature for 1 h. The resulting solid product was collected by vacuum filtration, washed with cold anhydrous diethyl ether (3 mL), and used without further purification for the next step. To a solution of the suitable acyl chloride (10 mmol) in anhydrous THF (20 mL), a solution of sodium hydroxide (NaOH) 2M was added dropwise, until complete alkalization, reaching a pH of 14. The reaction mixture was stirred at room temperature overnight. A solution of hydrochloric acid (HCl) 6M was added up to pH = 1. The resulting solid precipitate was collected by vacuum filtration, washed with H_2_O, and dried under vacuum for 24 h.

The spectroscopic data for compounds **16a**–**d** are in agreement with [25].

##### (5-Chloro-1-methyl-1H-indol-3-yl)-Oxo-Acetic Acid (**16e**)

Yield: 90%; Yellow solid; mp: 186.3 °C; ^1^H NMR (200 MHz, DMSO-*d_6_*) δ: 3.82 (3H, s), 7.16 (1H, dd, *J* = 8.3, 1.8 Hz), 7.32 (1H, d, *J* = 8.3 Hz), 8.23 (1H, d, *J* = 1.8 Hz), 8.46 (1H, s), 13.87 (1H, s); ^13^C{^1^H} NMR (50 MHz, DMSO-*d_6_*) δ: 33.5, 111.6, 117.1, 121.2, 125.7, 128.4, 131.6, 134.9, 135.5, 170.4, 173.5; Anal. Calculated for C_11_H_8_ClNO_3_ (MW: 237.64): C, 55.60; H, 3.39; N, 5.89%. Found: C, 55.68; H, 3.51; N, 5.95%.

#### 3.1.7. General Procedure for the Synthesis of 1,2,4-Oxadiazol Based-Topsentins **6a**–**e** and **7a**–**h**

The reaction between the two key building blocks **8**, **11**, **12** and **16a**–**e** was performed in DMF and in the presence of *N*-(3-dimethylaminopropyl)-*N*’-ethylcarbodiimide hydrochloride (EDC) and hydroxybenzotriazole (HOBt) as the coupling reagents, inducing the formation of an amide bond by previous activation of the carboxylic acid group. Subsequent in situ temperature-catalyzed cyclodehydration, warming the reaction mixture at 100 °C for 15 min, gave the desired oxadiazole derivatives **6a**–**e** and **7a**–**h** in yields ranging from 72–84%.

##### (5-Bromo-1-methyl-1H-indol-3-yl)(3-(thiophen-3-yl)-1,2,4-oxadiazol-5-yl)methanone (**6a**)

Yield: 72%; Yellow solid; mp: 206.7 °C; ^1^H NMR (200 MHz, DMSO-*d6*) δ: 4.00 (3H, s), 7.53 (1H, dd, *J* = 8.7, 2.0 Hz), 7.65 (1H, d, *J* = 8.6 Hz), 7.74 (1H, dd, *J* = 5.0, 1.2 Hz), 7.88–7.81 (1H, m), 8.41 (1H, d, *J*= 2.0 Hz), 8.56 (1H, dd, *J* = 3.0, 1.2 Hz), 9.06 (1H, s); ^13^C{^1^H} NMR (50 MHz, DMSO-*d_6_*) δ: 34.4, 112.5, 114.2, 117.0, 124.0, 126.3, 127.2, 127.5, 128.5, 129.3, 130.2, 136.9, 143.7, 165.1, 170.4, 170.9; Anal. Calculated for C_16_H_10_BrN_3_O_2_S (MW: 388.24): C, 49.50; H, 2.60; N, 10.82%. Found: C, 49.54; H, 2.64; N, 10.86%.

##### (5-Methoxy-1-methyl-1H-indol-3-yl)(3-(thiophen-3-yl)-1,2,4-oxadiazol-5-yl)methanone (**6b**)

Yield: 82%; Yellow solid; mp: 189.2 °C; ^1^H NMR (200 MHz, DMSO-*d_6_*) δ: 3.84 (3H, s), 3.97 (3H, s), 7.01 (1H, dd, *J* = 8.9, 2.5 Hz), 7.56 (1H, d, *J* = 8.9 Hz), 7.74 (1H, dd, *J* = 5.1, 1.2 Hz), 7.80 (1H, d, *J* = 2.5 Hz), 7.84 (1H, dd, *J* = 5.1, 3.0 Hz), 8.85 (1H, dd, *J* = 3.0, 1.2 Hz), 8.94 (1H, s); ^13^C{^1^H} NMR (50 MHz, DMSO-*d_6_*) δ: 34.4, 55.9, 104.1, 112.8, 112.9, 113.9, 126.3, 127.6, 127.9, 129.3, 130.1, 132.9, 142.7, 157.3, 165.1, 170.2, 170.3; Anal. Calculated for C_17_H_13_N_3_O_3_S (MW: 339.37): C,60.17; H, 3.86; N, 12.38%. Found: C, 60.25; H, 3.69; N, 12.51%.

##### (5-Fluoro-1-methyl-1H-indol-3-yl)(3-(thiophen-3-yl)-1,2,4-oxadiazol-5-yl)methanone (**6c**)

Yield: 74%; Dark yellow solid; mp: 100.8 °C; ^1^H NMR (200 MHz, DMSO-*d_6_*) δ: 4.01 (3H, s), 7.24 (1H, td, *J* = 9.2, 8.9, 2.6 Hz), 7.71 (1H, dd, *J* = 8.9, 4.4 Hz), 7.75 (1H, dd, *J* = 5.1, 1.2 Hz), 7.85 (1H, dd, *J* = 5.0, 3.0 Hz), 7.97 (1H, dd, *J* = 9.2, 2.6 Hz), 8.56 (1H, dd, *J* = 3.0, 1.2 Hz), 9.09 (1H, s); ^13^C{^1^H} NMR (50 MHz, DMSO-*d_6_*) δ: 34.5, 107.1 (*J_C4-F_* = 25.4 Hz), 112.6 (*J_C6-F_* = 26.2 Hz), 113.0 (*J_C7a-F_* = 4.0 Hz), 113.6 (*J_C7-F_* = 9.6 Hz), 126.3, 127.5, 127.6 (*J_C3a-F_* = 11.2 Hz), 129.3, 130.1, 134.7, 144.0, 160.2 (*J_C5-F_ =* 237.1 Hz), 166.1, 170.3, 171.0; Anal. Calculated for C_16_H_10_FN_3_O_2_S (MW: 327.33): C,58.71; H, 3.08; N, 12.84%. Found: C, 58.74; H, 3.11; N, 12.87%.

##### (1-Methyl-1H-indol-3-yl)(3-(thiophen-3-yl)-1,2,4-oxadiazol-5-yl)methanone (**6d**)

Yield: 81%; Yellow solid; mp: 178.3 °C; ^1^H NMR (200 MHz, DMSO-*d_6_*) δ: 4.01 (3H, s), 7.24–7.51 (2H, m), 7.65–7.68 (1H, m), 7.75 (1H, dd, *J* = 5.1, 1.2 Hz), 7.85 (1H, dd, *J* = 5.0, 3.0 Hz), 8.30–8.35 (1H, m), 8.56 (1H, dd, J = 3.0, 1.2 Hz), 9.04 (1H, s); ^13^C{^1^H} NMR (50 MHz, DMSO-*d_6_*) δ: 34.2, 111.9, 113.1, 122.0, 124.1, 124.6, 126.4, 126.8, 127.5, 129.4, 130.1, 138.0, 143.0, 165.1, 170.4, 171.2; Anal. Calculated for C_16_H_11_N_3_O_2_S (MW: 309.34): C, 62.12; H, 3.58; N, 13.58%. Found: C, 62.08; H, 3.54; N, 13.54%.

##### (5-Chloro-1-methyl-1H-indol-3-yl)(3-(thiophen-3-yl)-1,2,4-oxadiazol-5-yl)methanone (**6e**)

Yield: 80%; Yellow solid; mp: 193.8 °C; ^1^H NMR (200 MHz, DMSO-*d_6_*) δ: 4.01 (3H, s), 7.42 (1H, dd, *J* = 8.7, 2.1 Hz), 7.71 (1H, d, *J* = 8.7 Hz), 7.75 (1H, dd, *J* = 5.1, 1.2 Hz), 7.85 (1H, dd, *J* = 5.1, 3.0 Hz), 8.26 (1H, d, *J* = 2.1 Hz), 8.56 (1H, dd, *J* = 3.0, 1.2 Hz), 9.08 (s, 1H); ^13^C{^1^H} NMR (50 MHz, DMSO-*d_6_*) δ: 34.4, 112.6, 113.8, 121.0, 124.6, 126.3, 127.5, 128.0, 128.9, 129.3, 130.2, 136.6, 143.9, 165.1, 170.4, 170.9; Anal. Calculated for C_16_H_10_ClN_3_O_2_S (MW: 343.79): C, 55.90; H, 2.93; N, 12.22%. Found: C, 55.87; H, 2.90; N, 12.19%.

##### (3-(1H-Pyrrol-2-yl)-1,2,4-oxadiazol-5-yl)(5-fluoro-1-methyl-1H-indol-3-yl)methanone (**7a**)

Yield: 76%; Yellow solid; mp: 203.7 °C; ^1^H NMR (200 MHz, DMSO-*d_6_*) δ: 4.01 (3H, s), 6.30 (1H, dd, *J* = 3.6, 2.4 Hz), 6.99 (1H, dd, *J* = 3.6, 1.6 Hz), 7.13 (1H, dd, *J* = 4.1, 2.1 Hz), 7.26 (1H, td*, J* = 9.2, 9.0, 2.6 Hz), 7.71 (1H, dd, *J* = 9.0, 4.4 Hz), 7.97 (1H, dd, *J* = 9.2, 2.6 Hz), 9.11 (1H, s), 12.06 (1H, s); ^13^C{^1^H} NMR (50 MHz, DMSO-*d_6_*) δ: 34.5, 107.1 (*J_C4-F_* = 25.1 Hz), 110.4, 112.6 (*J_C6-F_* = 25.9 Hz), 112.9, 113.0 (*J_C7a-F_* = 3.8 Hz), 113.5 (*J_C7-F_* = 9.9 Hz), 117.8, 123.7, 127.6 (*J_C3a-F_* = 11.0 Hz), 134.7, 144.0, 160.1 (*J_C5-F_* = 237.3 Hz), 161.3, 163.2, 170.4; Anal. Calculated for C_16_H_11_FN_4_O_2_ (MW: 310.28): C, 61.93; H, 3.57; N, 18.06%. Found: C, 61.91; H, 3.44; N, 18.12%.

##### (3-(1H-Pyrrol-2-yl)-1,2,4-oxadiazol-5-yl)(5-bromo-1-methyl-1H-indol-3-yl)methanone (**7b**)

Yield: 78%; Yellow solid; mp: 219.1 °C; ^1^H NMR (200 MHz, DMSO-*d_6_*) δ: 4.00 (3H, s), 6.30 (1H, dt, *J* = 3.6, 2.4 Hz), 6.99 (1H, ddd, *J* = 3.8, 2.4, 1.5 Hz), 7.13 (1H, td, *J* = 2.7, 1.5 Hz), 7.55 (1H, dd, *J* = 8.7, 2.0 Hz), 7.67 (1H, d, *J* = 8.7 Hz), 8.42 (1H, d, *J* = 2.0 Hz), 9.10 (1H, s), 12.06 (1H, s); ^13^C{^1^H} NMR (50 MHz, DMSO-*d_6_*) δ: 34.4, 110.4, 112.5, 112.9, 114.2, 116.9, 117.8, 123.7, 124.0, 127.1, 128.6, 136.8, 143.8, 156.1, 170.3, 170.5; Anal. Calculated for C_16_H_11_BrN_4_O_2_ (MW: 371.19): C, 51.77; H, 2.99; N, 15.09%. Found: C, 51.80; H, 3.02; N, 15.12%.

##### (3-(1H-Pyrrol-2-yl)-1,2,4-oxadiazol-5-yl)(5-methoxy-1-methyl-1H-indol-3-yl)methanone (**7c**)

Yield: 83%; Brown solid; mp: 205.2 °C; ^1^H NMR (200 MHz, DMSO-*d_6_*) δ: 3.84 (3H, s), 3.97 (3H, s), 6.30 (1H, m), 6.98 (1H, m), 7.02 (1H, dd, *J* = 8.9, 2.5 Hz), 7.12–7.14 (1H, m), 7.58 (1H, d, *J* = 8.7 Hz), 7.81 (1H, d, *J* = 2.0 Hz), 8.98 (1H, s), 12.06 (1H, s); ^13^C{^1^H} NMR (50 MHz, DMSO-*_d6_*) δ: 34.4, 55.9, 104.1, 110.4, 112.8, 112.9, 112.9, 113.9, 117.8, 123.7, 127.9, 132.9, 142.8, 157.3, 163.1, 170.3, 170.7; Anal. Calculated for C_17_H_14_N_4_O_3_ (MW: 322.32): C, 63.35; H, 4.38; N, 17.38%. Found: C, 63.39; H, 4.42; N, 17.42%.

##### (3-(1H-Pyrrol-2-yl)-1,2,4-oxadiazol-5-yl)(1-methyl-1H-indol-3-yl)methanone (**7d**)

Yield: 79%; Yellow solid; mp: 170.8 °C; ^1^H NMR (200 MHz, DMSO-*d_6_*) δ: 4.00 (3H, s), 6.30 (1H, dt, *J* = 3.6, 2.3 Hz), 6.99 (1H, dt, *J* = 3.7, 1.9 Hz), 7.13 (1H, dd, *J* = 4.1, 2.6 Hz), 7.34–7.44 (2H, m), 7.67 (1H, dd, *J* = 6.8, 1.7 Hz), 8.29–8.33 (1H, m), 9.06 (1H, s), 12.07 (1H, s); ^13^C{^1^H} NMR (50 MHz, DMSO-*d_6_*) δ: 34.2, 110.4, 111.9, 112.8, 113.2, 117.8, 122.0, 123.7, 124.1, 124.6, 126.9, 138.0, 143.1, 163.1, 170.5, 170.7; Anal. Calculated for C_16_H_12_N_4_O_2_ (MW: 292.29): C, 65.75; H, 4.14; N, 19.17%. Found: C, 65.76; H, 4.15; N, 19.18%.

##### (3-(1H-Pyrrol-2-yl)-1,2,4-oxadiazol-5-yl)(5-chloro-1-methyl-1H-indol-3-yl)methanone (**7e**)

Yield: 80%; Yellow solid; mp: 227.7 °C; ^1^H NMR (200 MHz, DMSO-*d_6_*) δ: 4.01 (3H, s), 6.30 (1H, dt, *J* = 3.6, 2.4 Hz), 6.99 (1H, ddd, *J* = 3.8, 2.5, 1.5 Hz), 7.13 (1H, td, *J* = 2.7, 1.5 Hz), 7.43 (1H, dd, *J* = 8.7, 2.1 Hz), 7.72 (1H, d, *J* = 8.7 Hz), 8.27 (1H, d, *J* = 2.1 Hz), 9.12 (1H, s), 12.07 (1H, s); ^13^C{^1^H} NMR (50 MHz, DMSO-*d_6_*) δ: 34.5, 110.4, 112.6, 112.9, 113.8, 117.8, 121.0, 123.7, 124.6, 128.0, 128.9, 136.6, 144.0, 163.2, 170.3, 170.5; Anal. Calculated for C_16_H_11_ClN_4_O_2_ (MW: 326.74): C, 58.82; H, 3.39; N, 17.15%. Found: C, 58.80; H, 3.37; N, 17.13%.

##### (5-Fluoro-1-methyl-1H-indol-3-yl)(3-(1-methyl-1H-pyrrol-2-yl)-1,2,4-oxadiazol-5-yl)methanone (**7f**)

Yield: 83%; Yellow solid; mp: 172.7 °C; ^1^H NMR (200 MHz, DMSO-*d_6_*) δ: 3.98 (3H, s), 4.01 (3H, s), 6.24 (1H, dd, *J* = 3.8, 2.6 Hz), 7.10 (1H, dd, *J* = 3.8, 1.8 Hz), 7.17 (1H, dd, *J* = 4.1, 2.1 Hz), 7.27 (1H, td, *J* = 9.2, 9.0, 2.6 Hz), 7.71 (1H, dd, *J* = 9.0, 4.4 Hz), 7.97 (1H, dd, *J* = 9.2, 2.6 Hz), 9.00 (1H, s); ^13^C{^1^H} NMR (50 MHz, DMSO-d_6_) δ: 34.5, 37.2, 107.1 (d, *J_C4-F_* = 25.6 Hz), 109.1, 112.6 (d, *J_C6-F_* = 25.8 Hz), 113.0 (s, *J_C7a-F_* = 3.7 Hz), 113.6 (d, *J_C7-F_* = 9.7 Hz), 116.0, 119.5, 125.1, 127.5 (s, *J_C3a-F_* = 15.6 Hz), 129.6, 134.7, 143.8, 160.1 (s, *J_C5-F_* = 237.3 Hz), 163.1, 170.5; Anal. Calculated for C_17_H_13_FN_4_O_2_ (MW: 324.31): C, 62.96; H, 4.04; N, 17.28%. Found: C, 62.82; H, 4.10; N, 17.14%.

##### (1-Methyl-1H-indol-3-yl)(3-(1-methyl-1H-pyrrol-2-yl)-1,2,4-oxadiazol-5-yl)methanone (**7g**)

Yield: 78%; Yellow solid; mp: 152.5 °C; ^1^H NMR (200 MHz, DMSO-*d_6_*) δ: 3.98 (3H, s), 3.99 (3H, s), 6.24 (1H, dt, *J* = 3.5, 2.8 Hz), 7.09 (1H, dd, *J* = 3.8, 1.7 Hz), 7.16–7.17 (1H, m), 7.37–7.40 (2H, m), 7.66 (1H, dd, *J* = 6.9, 1.7 Hz), 8.30 (1H, dd, *J* = 6.6, 1.8 Hz), 8.93 (1H, s); ^13^C{^1^H} NMR (50 MHz, DMSO-*d_6_*) δ: 34.2, 37.1, 109.0, 111.3, 111.8, 112.3, 113.1, 115.8, 119.6, 121.5, 121.9, 124.0, 124.6, 129.5, 142.8, 167.3, 170.5; Anal. Calculated for C_17_H_14_N_4_O_2_ (MW: 306.32): C, 66.66; H, 4.61; N, 18.29%. Found: C, 66.78; H, 4.67; N, 18.20%.

##### (5-Methoxy-1-methyl-1H-indol-3-yl)(3-(1-methyl-1H-pyrrol-2-yl)-1,2,4-oxadiazol-5-yl)methanone (**7h**)

Yield: 84%; Yellow solid; mp: 165.6 °C; ^1^H NMR (200 MHz, DMSO-*d_6_*) δ: 3.84 (3H, s), 3.96 (3H, s), 3.98 (3H, s), 6.24 (1H, dd, *J* = 3.8, 2.6 Hz), 7.02 (1H, dd, *J* = 8.9, 2.5 Hz), 7.08 (1H, dd, *J* = 3.8, 1.8 Hz), 7.16 (1H, m), 7.57 (1H, d, *J* = 8.7 Hz), 7.81 (1H, d, *J* = 2.5 Hz), 8.86 (1H, s); ^13^C{^1^H} NMR (50 MHz, DMSO-*d_6_*) δ: 34.4, 37.2, 55.9, 104.1, 109.0, 112.8, 112.9, 113.9, 115.9, 119.5, 127.8, 129.6, 132.9, 142.5, 157.3, 163.1, 170.2, 170.3; Anal. Calculated for C_18_H_16_N_4_O_3_ (MW: 336.34): C, 64.28; H, 4.79; N, 16.66%. Found: C, 64.26; H, 4.77; N, 16.64%.

### 3.2. Biology

#### 3.2.1. Drugs and Chemicals

The synthesized derivatives **6** and **7** were dissolved in DMSO. The culture medium, fetal bovine serum (FBS), penicillin (50 IU/mL), and streptomycin (50 mg/mL) were obtained from Gibco (Gaithersburg, MD, USA). All other reagents were procured from Sigma (Zwijndrecht, The Netherlands).

#### 3.2.2. Cell Culture

The cells were cultured in RPMI-1640 (Roswell Park Memorial Institute 1640) with 10% heat-inactivated FBS and 1% penicillin/streptomycin, or in DMEM (Dulbecco’s Modified Eagle’s Medium) supplemented with 10% heat-inactivated FBS and 1% HEPES. They were maintained at 37 °C in a humidified atmosphere containing 5% CO_2_ and 95% air and harvested using trypsin-EDTA. For the wound healing assay, PDAC cell lines, including Patu-T, Suit-2, Capan-1, and Panc-1, were selected. The Suit-2 cells were kindly provided by Dr. A Frampton (Imperial College, UK) while all the other cells were purchased from the ATCC (American Type Culture Collection) (Manassas, VA, USA) and were tested for their authentication by STR–PCR (Short Tandem Repeat-Polymerase Chain Reaction). Additionally, all these cells were routinely tested for mycoplasma.

However, some cell lines, such as Capan-1 and MIA PaCa-2, showed no significant pro-apoptotic or necrotic effects after 24 h of compound exposure, which did not enable a consistent analysis of the experimental results and they were excluded in the following analyses.

#### 3.2.3. Viability Assay In Vitro

The cytotoxic activity of the 1,2,4-oxadiazol-based topsentins (**6a**–**e** and **7a**–**h**) against differentiated pancreatic cancer cell lines was assessed using the SRB chemosensitivity assay, as described previously [14]. Cells were seeded in triplicate into 96-well flat-bottom plates at a density of 3 × 103 cells/well in a total volume of 100 µL per well (for Suit-2, Panc-1, and Patu-T cell lines) and incubated at 37 °C for 24 h to allow the formation of a confluent monolayer. Following incubation, 100 µL of the test 1,2,4-oxadiazol-based topsentins (**6a**–**e** and **7a**–**h**) compounds, dissolved in DMSO at nine screening concentrations (from 0.1 μM to 40 μM), were added to the wells. The cells were then incubated for 72 h under controlled conditions (37 °C, 5% CO_2_, and 100% humidity).

At the end of the treatment period, the cells were fixed with 25 µL of 50% cold trichloroacetic acid (TCA) and kept at 4 °C for at least 60 min. The plates were then emptied, gently washed with deionized water, and air-dried at room temperature (RT) overnight. Next, the cells were stained with 50 µL of 0.4% SRB solution prepared in 1% acetic acid for 15 min at RT. Excess SRB stain was removed by gently washing the plates with 1% acetic acid, followed by drying at RT overnight. The bound SRB dye was solubilized using 150 µL of tris(hydroxymethyl)aminomethane (TRIS) buffer solution (pH 8.8). The plates were kept for four minutes in a microplate shaker before reading. Absorbance was measured at 490 nm using a microplate reader. Cell growth inhibition was calculated as the percentage of drug-treated cells relative to vehicle-treated control cells (“untreated cells or control”) OD (corrected for OD before drug addiction, “day-0”). The percentage of cell growth inhibition was determined using the following equation:

Cell Growth Inhibition (%) = [(mean ODcompound − mean ODday zero plate)/mean (ODcontrol cells − mean ODday zero plate)] × 100

The results were corrected using the day-zero plate (wells with cells cultured for only 24 h) and normalized to the control wells (untreated cells) to determine the percentage of viable cells. The 50% inhibitory concentration (IC_50_) was calculated through non-linear least squares curve fitting using GraphPad PRISM (Intuitive Software for Science, San Diego, CA, USA). According to the NCI protocol, IC_50_ is referred to as GI50 (50% growth inhibitory concentration). The data are presented as mean values ± SEM.

Initially, the cells were treated with three screening concentrations (0.1 µM, 1 µM and 10 µM) of each compound from series **7** for 72 h, and cell proliferation was evaluated using the SRB chemosensitivity assay. To further determine the IC_50_ values for each PDAC cell line, an SRB assay was performed after treating the cells with eight increasing concentrations (ranging from 0.1 µM to 40 µM) of compounds **7a**–**f** for 72 h. The antitumor activity of the compounds was quantified based on the IC_50_ values, representing the concentration of a compound needed to achieve 50% growth inhibition relative to the untreated control cells.

#### 3.2.4. Wound Healing Assay

Cell migration was evaluated using a wound healing assay. A total of 5 × 10^4^ cells per well for Patu-T and 6 × 10^4^ cells per well for Suit-2 were seeded in 96-well plates and incubated overnight at 37 °C in a humidified atmosphere of 5% CO_2_ and 95% air to allow the formation of a confluent monolayer. Gaps (scratches) were introduced in the confluent monolayer using a scratch tool. Detached cells were removed by gentle washing, and fresh medium was added to the wells. Subsequently, the cells were treated with compounds **7b**–**f** at a concentration of 10 µM.

Following treatment, the cells were maintained at 37 °C in 5% CO_2_ and 95% air. Wound closure was monitored at various time points (T = 0, 4 h, 8 h, 20 h, and 24 h) using phase-contrast microscopy. Images were captured immediately after the scratch (T = 0) and at each subsequent time point using the Universal Grab 6.3 software (DCILabs) connected to a Leica microscope equipped with a JAI TMC-1327 camera. The percentage of migration was calculated using the following equation:

Percentage of Migration % = [(Width of the wound at T = 0 − Width of the wound at T = X)/(Width of the wound at T = 0)] × 100

#### 3.2.5. Clonogenic Assay

The clonogenic assay was performed to evaluate the long-term proliferative capacity and survival of Patu-T cells following treatment with compounds **7b**, **7d**, and **7f**. The cells were seeded in a six-well plate at low concentration (3000 cells/well). After 24 h, the cells were treated with the respective IC_50_ concentrations. The medium containing the drugs was refreshed every 3–4 days. Colonies were fixed with 100% ethanol and stained with 0.5% crystal violet (*w*/*v*) for visualization.

#### 3.2.6. Apoptosis Assay

The Annexin V-FITC apoptosis assay was conducted to detect late apoptotic cells by measuring the externalization of phosphatidylserine on the outer membrane. The binding buffer (BBA) was freshly prepared before the experiment by mixing 10 mM Hepes (pH 7.4), 140 mM NaCl, and 12.5 mM CaCl_2_. Alternatively, a 10× BBA buffer from the BD Biosciences (Franklin Lakes, NJ, USA) kit was diluted to 1× with MilliQ water. Cells were seeded and treated with **7b**, **7d**, and **7f** compounds according to the experimental design. After treatment, the supernatant was aspirated from each well using a multichannel pipette or vacuum, followed by a gentle wash with 50 µL of PBS containing Ca^2+^ and Mg^2+^ to prevent cell detachment.

Next, staining was performed by adding 29.7 µL of BBA mixed with 0.3 µL of Annexin V-FITC (final concentration 1:100) to each well. The cells were incubated for 10 min at room temperature in the dark to allow Annexin V binding. Following incubation, the supernatant was carefully removed using a multichannel pipette to avoid disturbing the cells, and each well was washed with 200 µL of BBA. For the final reading, 100 µL of BBA was added to each well with propidium iodide (PI). The fluorescence was then measured using a plate reader set to an excitation wavelength of 485 nm and an emission wavelength of 535 nm. For microscope-based observation, the cells were immediately examined after adding the final BBA solution to avoid changes in morphology caused by prolonged incubation.

To normalize the fluorescence data based on cell numbers, an SRB assay was performed. After the fluorescence measurement, cells were fixed by adding 25 µL of TCA to each well, followed by a 1 h incubation at 4 °C. The wells were then washed with 100 µL of PBS, stained with SRB, and quantified to determine the number of cells in each well. The normalized data allowed for accurate comparison of the fluorescence signals, providing a reliable assessment of the apoptotic response in the treated cells.

#### 3.2.7. ADME Studies

The ADME predictions were performed using SwissADME prediction software [32]. The number of H-bond donors, H-bond acceptors, and rotatable bonds, as well as the bioavailability, GI absorption, and BBB permeation, were evaluated.

## 4. Conclusions

In the present study, we efficiently synthesized a new series of 1,2,4-oxadiazole derivatives that showed promising antiproliferative activities in vitro against a series of PDAC cells.

Compounds **7b**, **7d**, and especially **7f** consistently exhibited potent antiproliferative effects, with IC_50_ values in the low micromolar range, particularly against Patu-T cells, mirroring the activity profiles reported for related 1,2,4-oxadiazole derivatives such as compound **5**. In line with these findings, our compounds effectively inhibited clonogenic survival, with **7f** achieving an 83% reduction in colony formation, underscoring their capacity to suppress long-term tumor cell proliferation.

Importantly, these derivatives also impaired cell migration, a critical step in PDAC metastasis, as demonstrated by scratch wound healing assays. Compound **7f** reduced migration by approximately 80% in SUIT-2 cells and showed comparable antimigratory activity in Patu-T cells. Furthermore, apoptosis induction assays revealed that **7f** and its analogs significantly triggered programmed cell death, with a 5.2-fold increase in apoptotic Patu-T cells.

Collectively, these findings support the oxadiazole topsentin scaffold as a promising platform for the development of novel anticancer agents targeting multiple hallmarks of PDAC progression.

## Figures and Tables

**Table 1:** Yields of the synthesis of **6a**–**e** and **7a**–**h**.

Compound	R	R1	Yield%
**6a**	-	Br	72
**6b**	-	OCH_3_	82
**6c**	-	F	74
**6d**	-	H	81
**6e**	-	Cl	80
**7a**	H	F	76
**7b**	H	Br	78
**7c**	H	OCH_3_	83
**7d**	H	H	79
**7e**	H	Cl	80
**7f**	CH_3_	F	83
**7g**	CH_3_	H	78
**7h**	CH_3_	OCH_3_	84

## Data Availability

Data are contained within the article and Supplementary Materials.

## Supplementary Materials

The following supporting information can be downloaded at: https://www.mdpi.com/article/10.3390/md23080327/s1, Figures S1–S12: NMR spectra of compounds **7a**–**7f**.

## Abbreviations

The following abbreviations are used in this manuscript:

PDAC	Pancreatic ductal adenocarcinoma
SRB	Sulforhodamine B
BBA	Binding buffer
THF	Tetrahydrofuran
DMF	*N,N*-Dimethylformamide
