# Supplementary material for: New Marine-Inspired Oxadiazole Derivatives for Use Against Pancreatic Ductal Adenocarcinoma
Source: Mar Drugs. 2025 Aug 14;23(8):327. doi: 10.3390/md23080327 (PMC12387776; doi:10.3390/md23080327)
Supplement: Supplementary file 1 [file marinedrugs-23-00327-s001.zip › marinedrugs-3814750-supplementary.pdf]

marine drugs

Article

New Marine-Inspired Oxadiazole Derivatives for Use Against Pancreatic Ductal Adenocarcinoma

Camilla Pecoraro [1], Daniela Carbone [1,*], Fares Hezam Mohammed Al Ostoot [1,2], Mahrou Vahabi [2], Giulia Lencioni [3], Patrizia Diana [1], Elisa Giovannetti [2,3] and Barbara Parrino [1]

1 Dipartimento di Scienze e Tecnologie Biologiche Chimiche e Farmaceutiche (STEBICEF), Università Degli Studi di Palermo, Via Archirafi 32, 90123 Palermo, Italy; camilla.pecoraro@unipa.it (C.P.); fareshezammohammed.alostoot@unipa.it (F.H.M.A.O.); patrizia.diana@unipa.it (P.D.); barbara.parrino@unipa.it (B.P.)

2 Department of Medical Oncology, Cancer Center Amsterdam, Amsterdam UMC, VU University Medical Center (VUmc), De Boelelaan 1117, 1081 HV Amsterdam, The Netherlands; m.vahabi@amsterdamumc.nl (M.V.); e.giovannetti@amsterdamumc.nl (E.G.)

3 Cancer Pharmacology Laboratory, Fondazione Pisana per la Scienza, Via Ferruccio Giovannini 13, San Giuliano Terme, 56017 Pisa, Italy; g.lencioni@fpscience.it

* Correspondence: daniela.carbone@unipa.it

Academic Editor: Alexander V Kornienko

Received: 28 July 2025
Revised: 7 August 2025
Accepted: 10 August 2025
Published: 14 August 2025

Citation: Pecoraro, C.; Carbone, D.; Al Ostoot, F.H.M.; Vahabi, M.; Lencioni, G.; Diana, P.; Giovannetti, E.; Parrino, B. New Marine-Inspired Oxadiazole Derivatives for Use Against Pancreatic Ductal Adenocarcinoma. *Mar. Drugs* **2025**, *23*, 327. https://doi.org/10.3390/md23080327

Copyright: © 2025 by the authors. Licensee MDPI, Basel, Switzerland. This article is an open access article distributed under the terms and conditions of the Creative Commons Attribution (CC BY) license (https://creativecommons.org/licenses/by/4.0/).

**Content**	**Figures**
NMR spectra	S1-S12

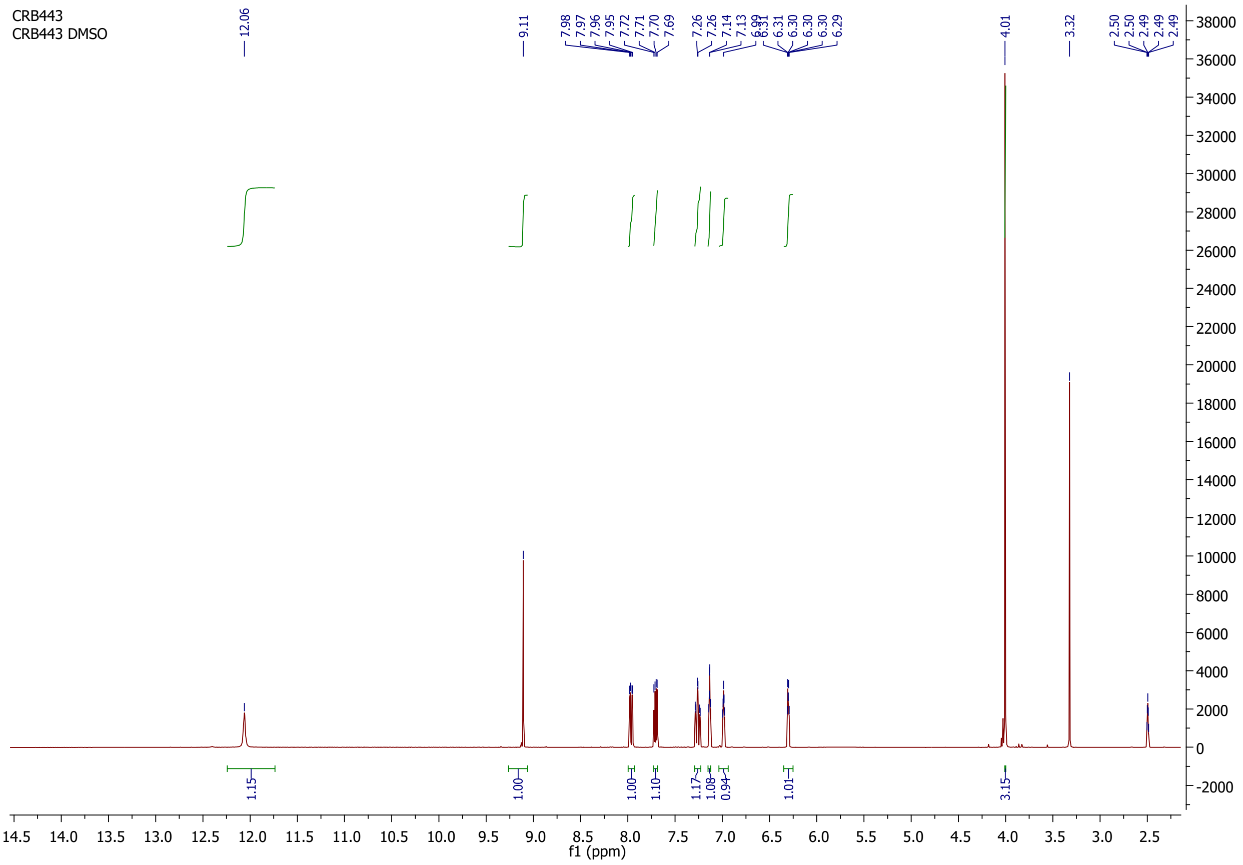

Figure S1. ^{1}H NMR spectrum (DMSO-d6) of compound **7a.**

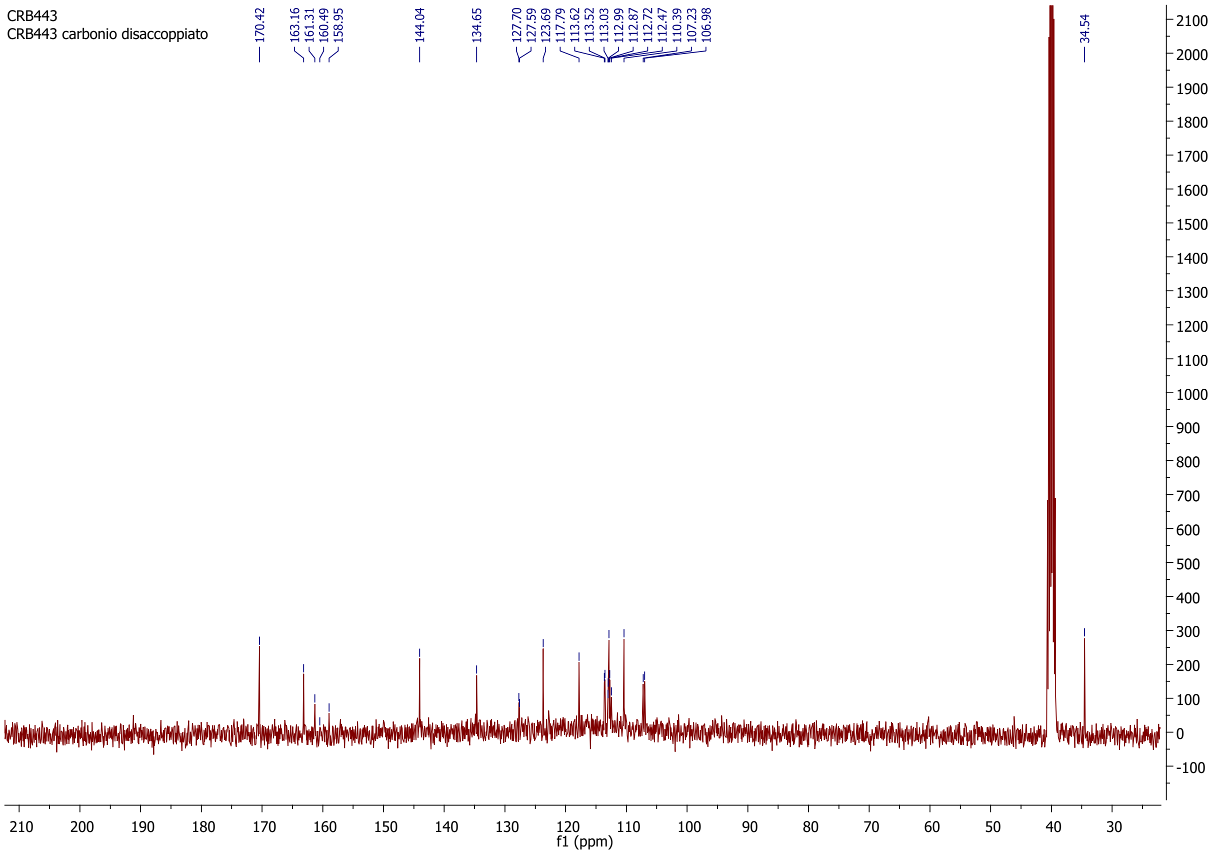

Figure S2. ^{13}C NMR spectrum (DMSO-d$_6$) of compound **7a.**

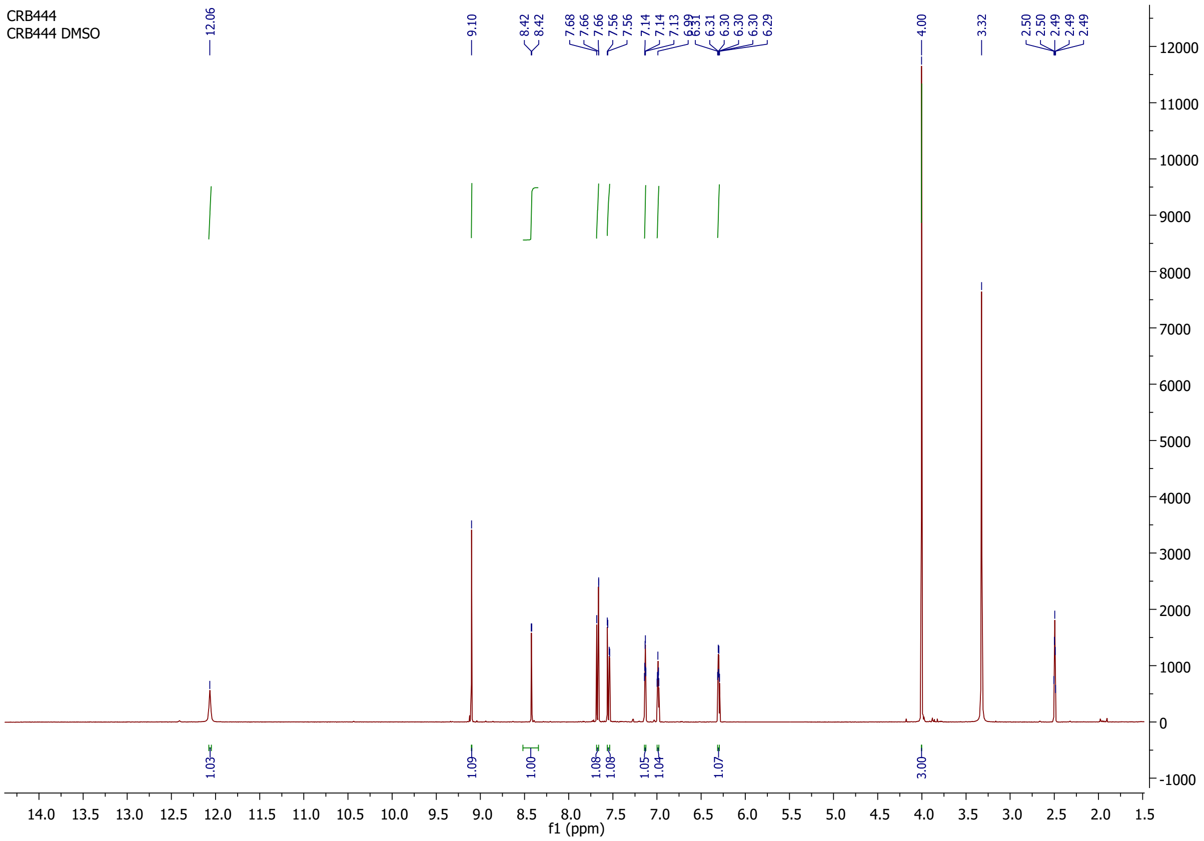

Figure S3. ^{1}H NMR spectrum (DMSO-d$_6$) of compound **7b.**

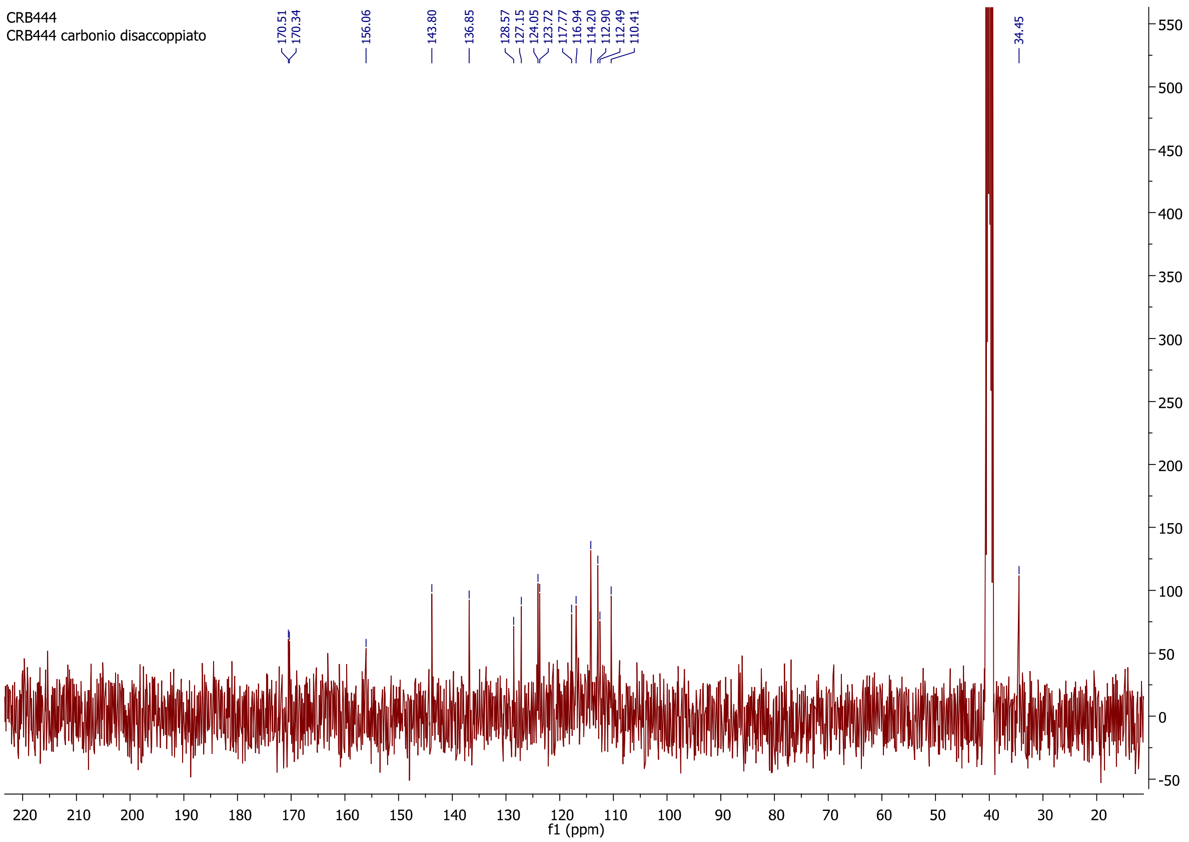

Figure S4. ^{13}C NMR spectrum (DMSO-d6) of compound **7b.**

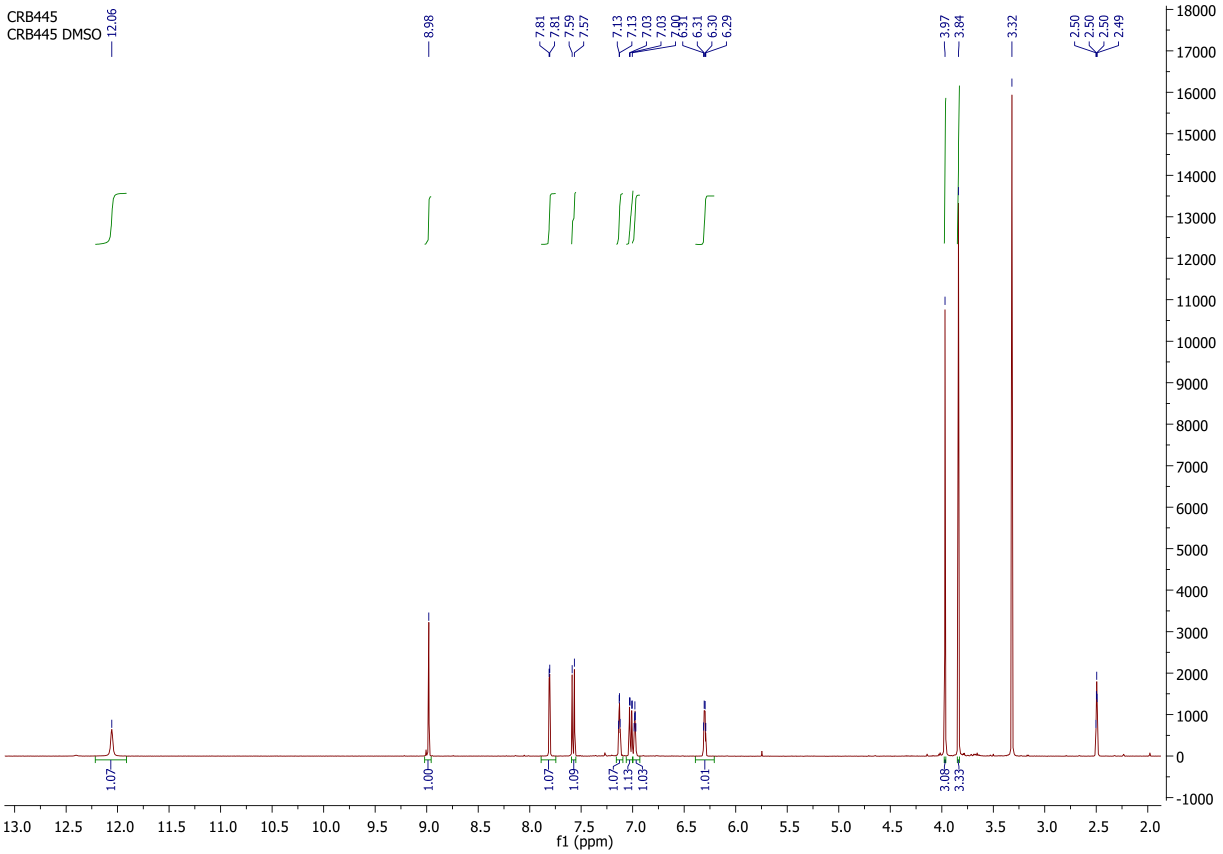

Figure S5. ^{1}H NMR spectrum (DMSO-d6) of compound **7c.**

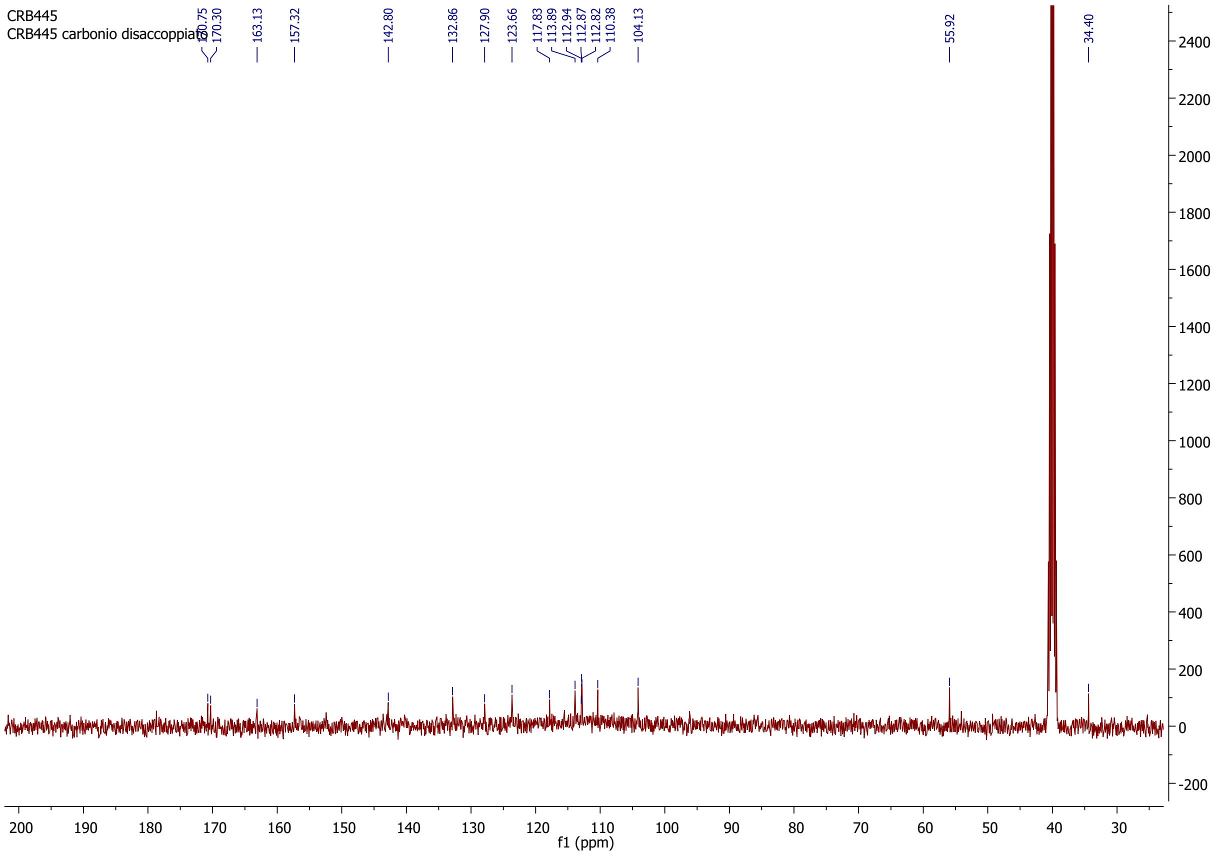

Figure S6. ^{13}C NMR spectrum (DMSO-d$_6$) of compound **7c.**

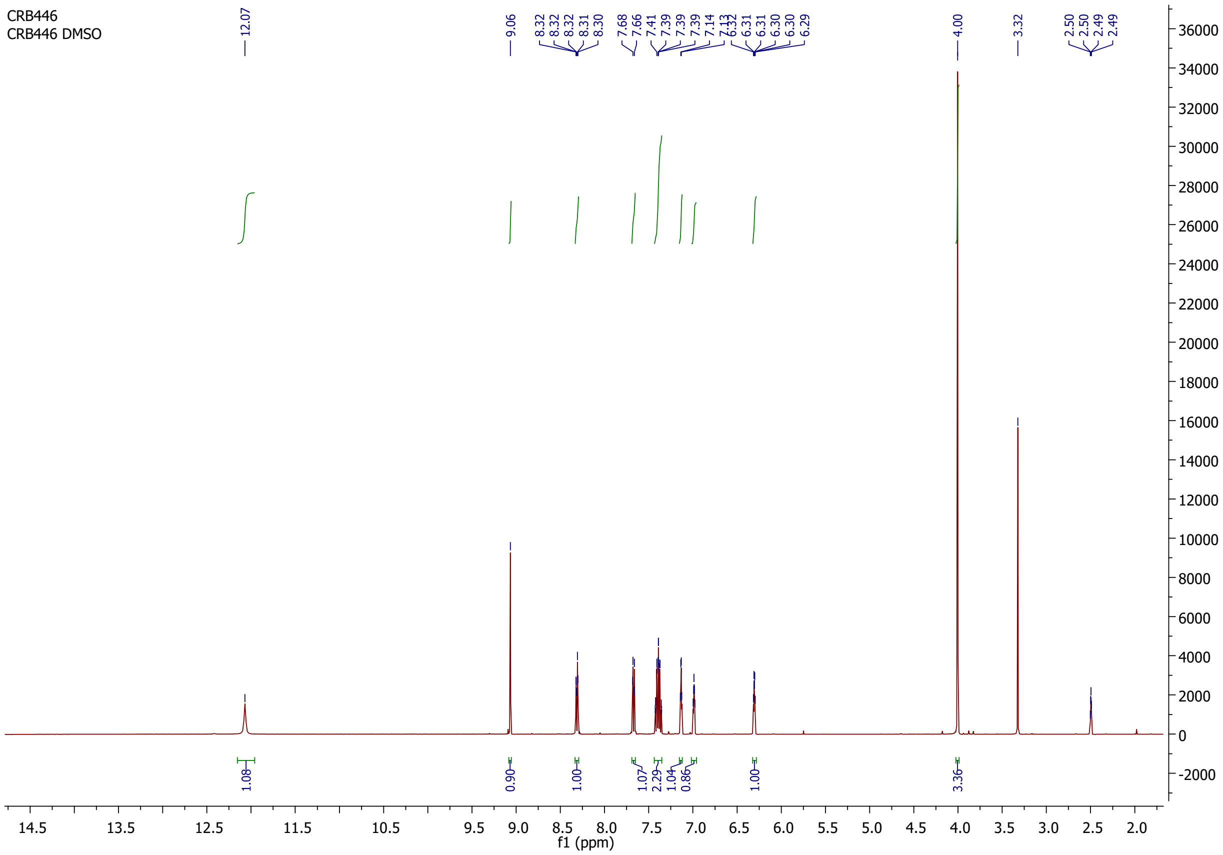

Figure S7. ^{1}H NMR spectrum (DMSO-d$_6$) of compound **7d.**

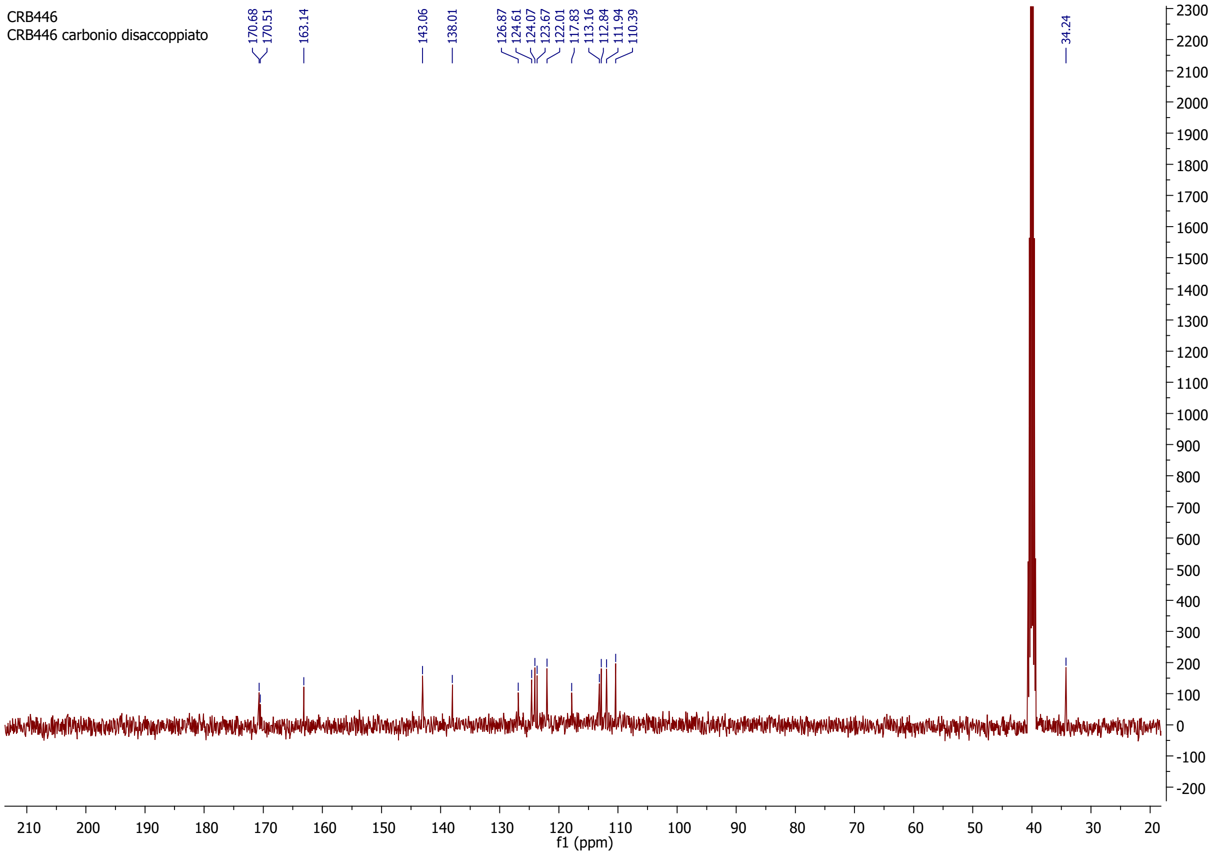

Figure S8. ^{13}C NMR spectrum (DMSO-d_6) of compound **7d.**

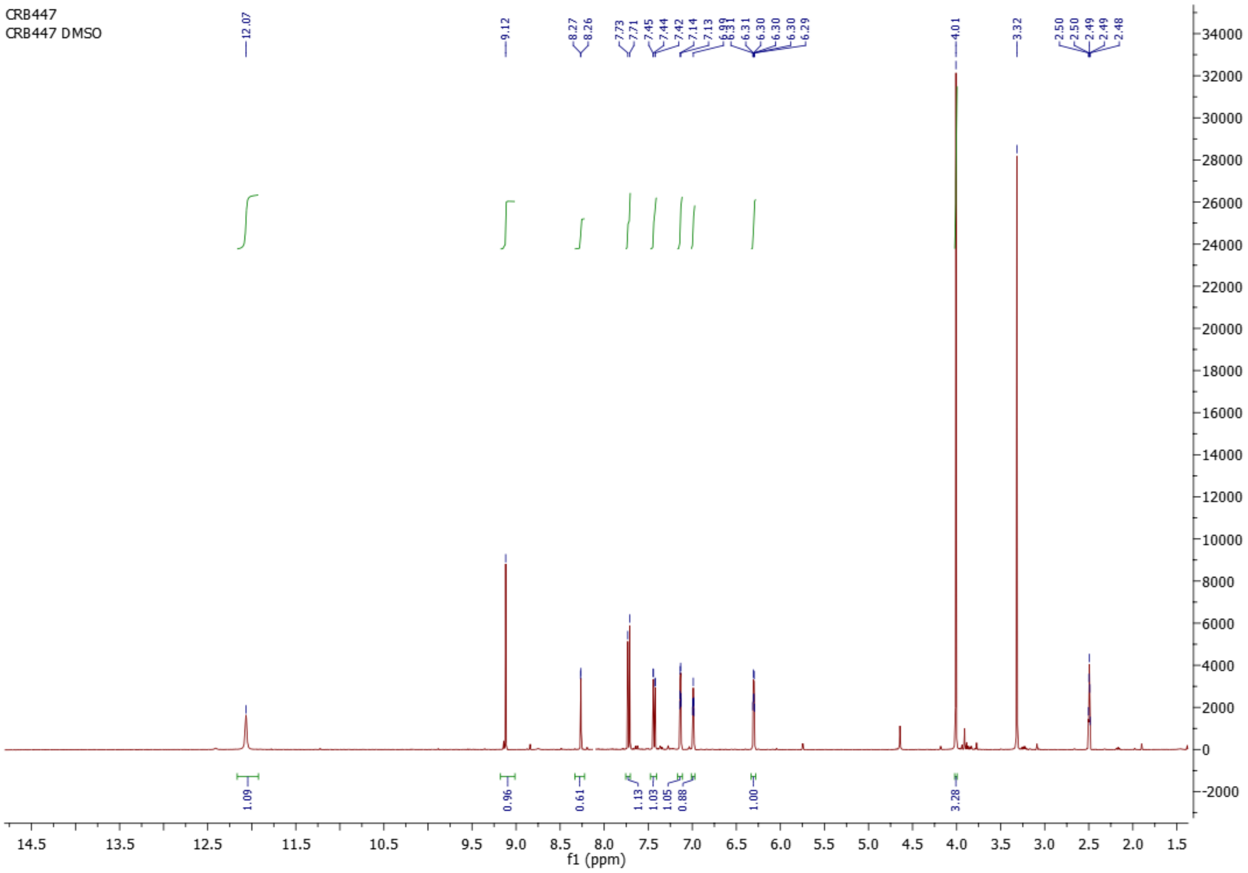

Figure S9. ^{1}H NMR spectrum (DMSO-d_6) of compound **7e.**

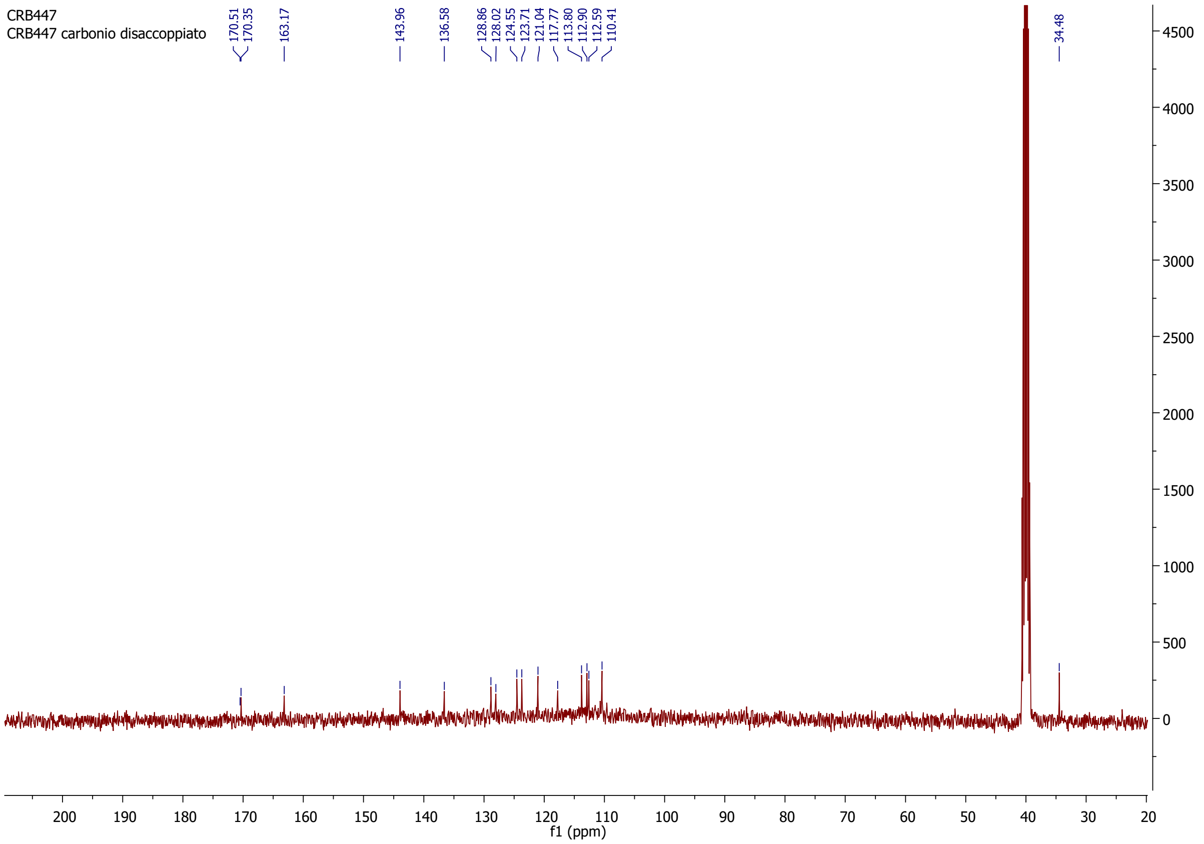

Figure S10. ^{13}C NMR spectrum (DMSO-d6) of compound **7e.**

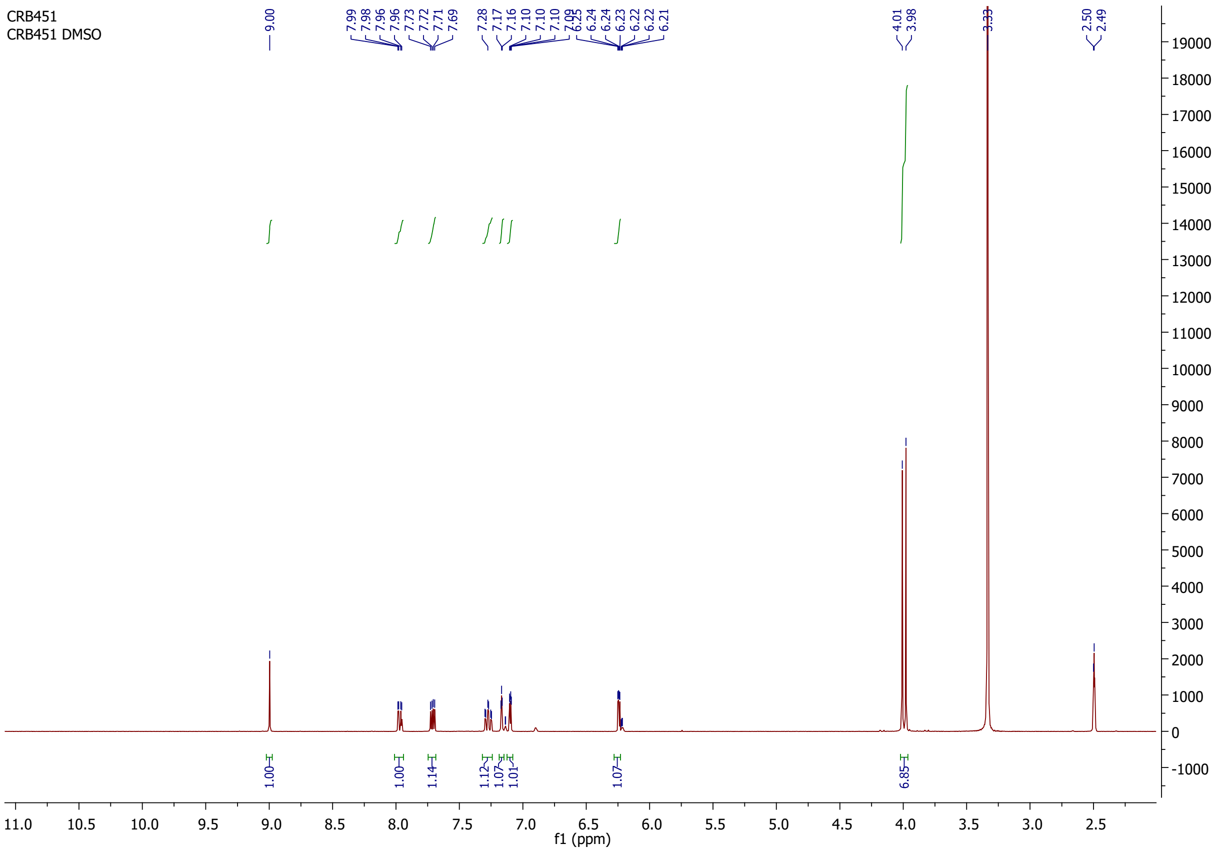

Figure S11. ^{1}H NMR spectrum (DMSO-d6) of compound **7f.**

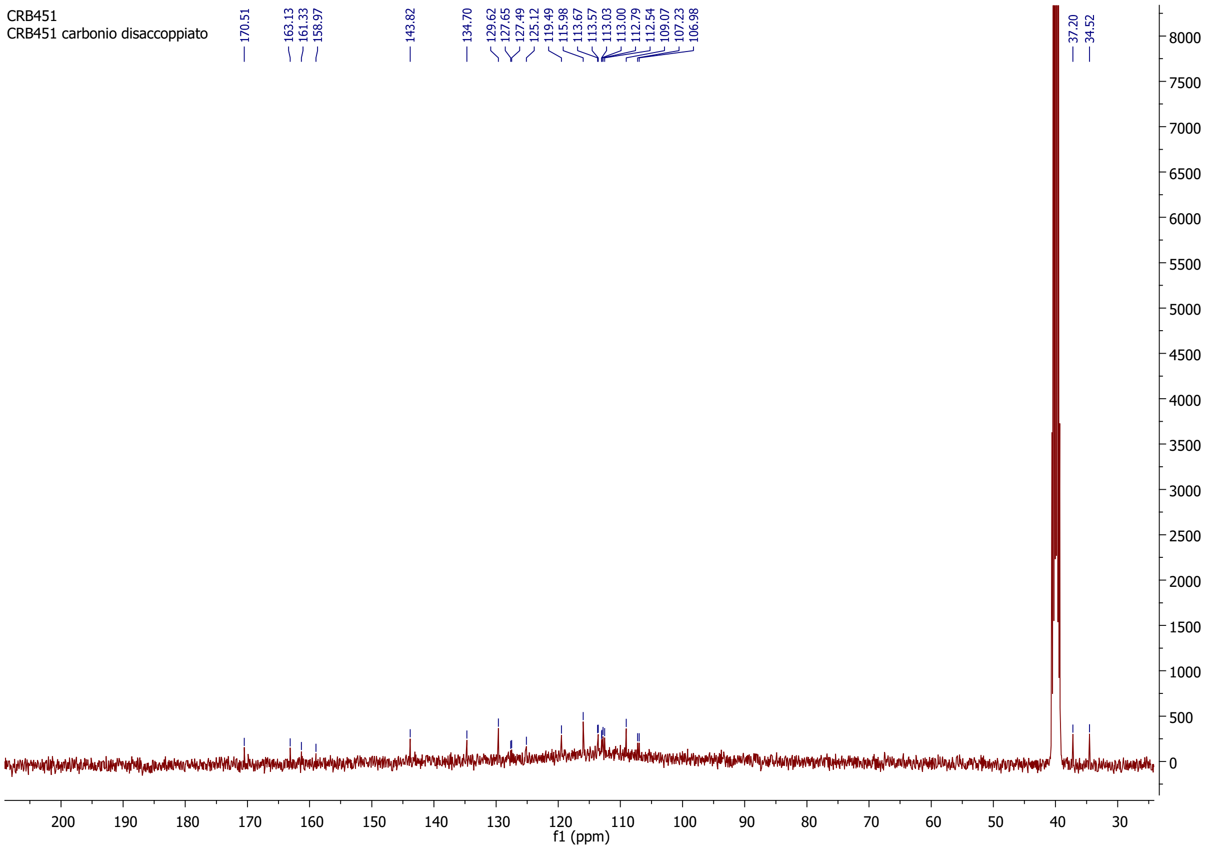

Figure S12. ^{13}C NMR spectrum (DMSO-d_6) of compound **7f.**
